# Berberine attenuates choline-induced atherosclerosis by inhibiting trimethylamine and trimethylamine-*N*-oxide production via manipulating the gut microbiome

**DOI:** 10.1038/s41522-021-00205-8

**Published:** 2021-04-16

**Authors:** Xingxing Li, Chunyan Su, Zhibo Jiang, Yuxin Yang, Yue Zhang, Mengxia Yang, Xiumin Zhang, Yu Du, Jin Zhang, Li Wang, Jiandong Jiang, Bin Hong

**Affiliations:** 1 NHC Key Laboratory of Biotechnology of Antibiotics, Institute of Medicinal Biotechnology, Chinese Academy of Medical Sciences & Peking Union Medical College, Beijing, China; 2 CAMS Key Laboratory of Synthetic Biology for Drug Innovation, Institute of Medicinal Biotechnology, Chinese Academy of Medical Sciences & Peking Union Medical College, Beijing, China

**Keywords:** Microbiome, Health care

## Abstract

Trimethylamine-*N*-oxide (TMAO), a derivative from the gut microbiota metabolite trimethylamine (TMA), has been identified to be an independent risk factor for promoting atherosclerosis. Evidences suggest that berberine (BBR) could be used to treat obesity, diabetes and atherosclerosis, however, its mechanism is not clear mainly because of its poor oral bioavailability. Here, we show that BBR attenuated TMA/TMAO production in the C57BL/6J and ApoE KO mice fed with choline-supplemented chow diet, and mitigated atherosclerotic lesion areas in ApoE KO mice. Inhibition of TMA/TMAO production by BBR-modulated gut microbiota was proved by a single-dose administration of d9-choline in vivo. Metagenomic analysis of cecal contents demonstrated that BBR altered gut microbiota composition, microbiome functionality, and *cutC*/*cntA* gene abundance. Furthermore, BBR was shown to inhibit choline-to-TMA conversion in TMA-producing bacteria in vitro and in gut microbial consortium from fecal samples of choline-fed mice and human volunteers, and the result was confirmed by transplantation of TMA-producing bacteria in mice. These results offer new insights into the mechanisms responsible for the anti-atherosclerosis effects of BBR, which inhibits commensal microbial TMA production via gut microbiota remodeling.

## Introduction

Atherosclerosis is the main pathological basis for cardiovascular disease, which is one of the leading causes of death worldwide^1^. While genetics have been shown to play a strong role in the development of atherosclerosis, environmental factors, such as diet and lifestyle, may also be a prominent contributor to the pathogenesis of atherosclerotic cardiovascular disease^2,3^. Gut microbiota that reside in the intestinal tract has been linked to numerous pathologies, including cardiovascular disease and metabolic syndrome^4–6^. For example, host cholesterol homeostasis, which includes modulating cholesterol levels, biosynthesis, and trafficking, is regulated by gut microbiota in addition to genetic and environmental factors^7^. The gut microbiota can convert diet nutrients into various small-molecule metabolites that enter the blood and can modulate host cardiovascular physiology^8–10^. In fact, some gut microbiota-derived metabolites have been associated with the progress of cardiovascular diseases. Short-chain fatty acids (SCFAs) are major products of gut microbial fermentation of nondigestible dietary fibers including acetate, propionate and butyrate, and may provide metabolic and cardiovascular benefits^11^. For example, with well-characterized anti-inflammatory and anti-oxidative properties^12,13^, butyrate was recently found to be anti-atherosclerosis by increasing ABCA1-mediated cholesterol efflux in peripheral macrophages^14^. Secondary bile acids, another kind of important gut microbiota metabolites, have been linked to lipid and glucose metabolism and prevention of atherosclerosis by functioning as signaling molecules^15–17^.

Trimethylamine-*N*-Oxide (TMAO), a gut microbiota-associated metabolite, has been reported to increase atherosclerosis risk, independent of known major atherosclerosis risk factors such as plasma low-density lipoprotein (LDL) cholesterol level and chronic inflammation^18–20^. In general, trimethylamine (TMA)-containing nutrients including choline, phosphatidylcholine and carnitine can be metabolized by several distinct gut microbial enzyme complexes including CutC/D and CntA/B to generate TMA^21,22^. TMA then enters the host circulation and is further metabolized to TMAO by host enzymes of the flavin monooxygenase (FMO) family in the liver^23^. In line with this notion, the production of TMA was proposed to be a pathway that might be therapeutically targeted at the level of gut microbiota. Several choline or betaine analogues were found or designed based on the ‘natural substrate’ mimicking strategy and characterized as specific CutC/D inhibitors. 3,3-dimethyl-1-butanol (DMB), betaine aldehyde and halomethylcholines showed TMA production inhibition from various human gut bacteria isolates containing *cutC/D* genes and human fecal suspensions. They could suppress plasma TMAO levels and reversed the choline diet-enhanced atherosclerosis and thrombus formation in mice^24–26^. Natural product resveratrol was also found to inhibit the choline metabolism by gut microbiota in mice, thus reduce the production of TMAO and retard the progression of atherosclerosis^27^.

Berberine (BBR) is an isoquinoline alkaloid extracted from herbal plants such as *Coptis chinensis* and *Berberis vulgaris*, which is traditionally used for the treatment of bacterial diarrhea in China. Its therapeutic effects in cardiovascular diseases and metabolic disorders have been clinically verified, and its molecular mechanisms of action have also been well illuminated^28–31^, e.g., BBR could reduce serum TDL and LDL cholesterol by stabilizing LDLR mRNA to post-transcriptionally increase LDLR expression^32^. However, with regard to its good therapeutic efficacy, the oral bioavailability of BBR is so poor that more than 99% of orally administrated BBR cannot be absorbed^33,34^. Like most traditional Chinese medicines^35^, BBR is usually administered orally and is inevitably exposed to the gut microbiota, thus BBR might also work through remodeling gut microbiota to reestablish host homeostasis. Some studies have shown that BBR may regulate the metabolism of the host at least partially via gut microbiota^36^ and their derived metabolites such as SCFAs^37^ and secondary bile acids^38^. Recently, Shi et al.^39^ showed that the anti-atherosclerotic effect of BBR on high-fat diet-fed ApoE KO mice was related to alterations in gut microbiota compositions and a lower serum TMAO level. However, the role of BBR in gut microbiota and its mode of action in decreasing choline-TMA-TMAO related atherosclerosis, independent of cholesterol homeostasis, are still largely unclear.

Given the close association among BBR, gut microbiota, TMAO and pathogenesis of atherosclerosis, the goal of this study is to examine the role of BBR in gut microbiota remodeling and TMA/TMAO generation in C57BL/6J and ApoE KO mice with atherosclerosis induced by choline-supplemented chow diet. We found that BBR decreased choline to TMA conversion and ultimately the serum TMAO level, and attenuated atherosclerosis development by remodeling gut microbiota through reducing functional gene levels of *cutC* and *cntA*, key genes of the main TMA-synthesis pathways. The inhibition of choline-to-TMA transformation by BBR was observed in some known TMA-producing bacteria cultured anaerobically in vitro and in choline-fed mice by transplantation of TMA-producing bacteria. Furthermore, BBR could reduce TMA production in the gut microbial consortium from feces of choline-fed mice and human volunteers, providing new evidences for the potential therapeutic value of BBR in treating atherosclerosis by modulating the gut microbiome.

## Results

### BBR decreased TMAO level in choline diet-fed C57BL/6J mice

**Fig. 1:**
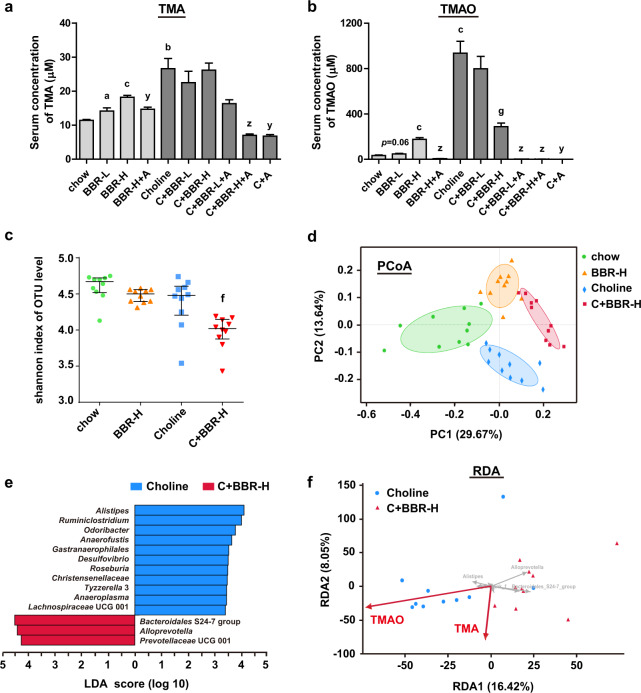
BBR decreased TMAO level in choline diet-fed C57BL/6J mice. Eight-week-old female C57BL/6J mice were fed chow, chow with 100 mg/kg BBR (BBR-L) or 200 mg/kg BBR (BBR-H), chow with choline (1%), or chow with choline (1%) plus 100 mg/kg BBR (C + BBR-L) or 200 mg/kg BBR (C + BBR-H) in the absence or presence of Abs (+A) for 6 weeks. **a**, **b** Serum TMA and TMAO levels were determined by HPLC/MS. Values are presented as means ± SEM (*n* = 10). a *p* < 0.05; b *p* < 0.01; c *p* < 0.001 (versus chow-diet group); g *p* < 0.001 (versus choline-diet grou*p*); y *p* < 0.01; z *p* < 0.001 (versus relevant group without Abs). **c** Microbiota alpha diversity was measured by 16S rRNA gene sequence analysis of the cecal content samples (*n* = 10 for each group) using Shannon index (based on OTU level). Error bars were median with interquartile ranges, and *p* values were from Kruskal–Wallis *H* test. f, *p* < 0.01 (versus choline-diet group). **d** Principal coordinate analysis (PCoA) of bray curtis distance was analyzed based on OTU level for microbiota beta diversity (*n* = 10 for each group, ANOSIM *R* = 0.6383, *p* = 0.001). **e** Linear discriminant analysis (LDA) identified the taxa most differentially abundant between the choline and C + BBR-H group at the genus level. Only taxa meeting an LDA significant threshold value of ≥3.3 are shown. **f** Redundancy analysis (RDA) visualized the correlation between key phylotypes of gut microbiota within choline and C + BBR-H group (at the genus level, gray arrows) and TMA and TMAO level (red arrows; correlation coefficient *r*^2^ = 0.3297, *p* = 0.042 and *r*^2^ = 0.3838, *p* = 0.013 respectively).

In order to investigate the effect of BBR on TMAO production from choline, C57BL/6J mice were fed chow diet or choline (1%) supplemented chow diet with or without BBR treatment. The body weight and food intake were similar between the groups. As shown in Fig. 1a, b, the serum TMA and TMAO levels in chow diet-fed mice were 11.4 µM and 32.7 µM. Compared with that, 6-weeks choline treatment significantly increased both serum TMA and TMAO levels by 2.3 and 28.6 folds, respectively, which were abolished by antibiotics (Abs) treatment. These findings are consistent with the previous reports that gut microbiota plays a key role in the production of TMA from choline^25^. Of note, 200 mg/kg of BBR-treatment for 6 weeks significantly reversed the increase of TMAO induced by choline (Fig. 1b), although the inhibitory effect on TMA level was not significant at that point (Fig. 1a). Interestingly, although still drastically lower than that in choline-fed mice, both serum TMA and TMAO levels in chow diet-fed mice were dose-dependently increased by BBR, which was also inhibited by Abs treatment (Fig. 1a, b).

To investigate whether TMAO inhibition by BBR under choline condition is mediated by gut microbial remodeling, we first explored bacterial populations in the cecal content samples from four groups of mice (chow, BBR-H, Choline, C + BBR-H) by 16S ribosomal RNA (rRNA) gene sequence analysis (Supplementary Fig. 1a). Compared to choline-fed mice, alpha diversity was significantly reduced in the C + BBR-H group as measured using the Shannon index (Fig. 1c) as well as the Sobs, Chao, and Simpson index (Supplementary Fig. 1b). The overall structural changes of the gut microbiota were then analyzed via principal coordinate analysis (PCoA). The result showed a dramatic difference in microbiota composition among the four groups at OTU level (Fig. 1d). Based on the linear discriminant analysis (LDA), some bacteria were significantly enriched in choline-fed mice, while others were enriched in BBR-treated mice (Fig. 1e). In order to investigate if the microbial composition changes correlated with the BBR-induced decrease of TMAO levels, we performed a redundancy analysis (RDA) (Fig. 1f). Most samples in the choline-fed mice (8/10) were positively correlated to TMAO levels, and a negative correlation was shown in the BBR-treated mice (10/10), suggesting that decreasing TMAO level by BBR under choline diet was directly associated with gut microbiota changes. The above results indicated that a high-choline diet coupled with BBR treatment could induce gut microbiota composition changes in C57BL/6J mice, which were directly related to the TMAO level.

### BBR decreased TMAO production and protected ApoE KO mice from TMAO-induced atherosclerosis

**Fig. 2:**
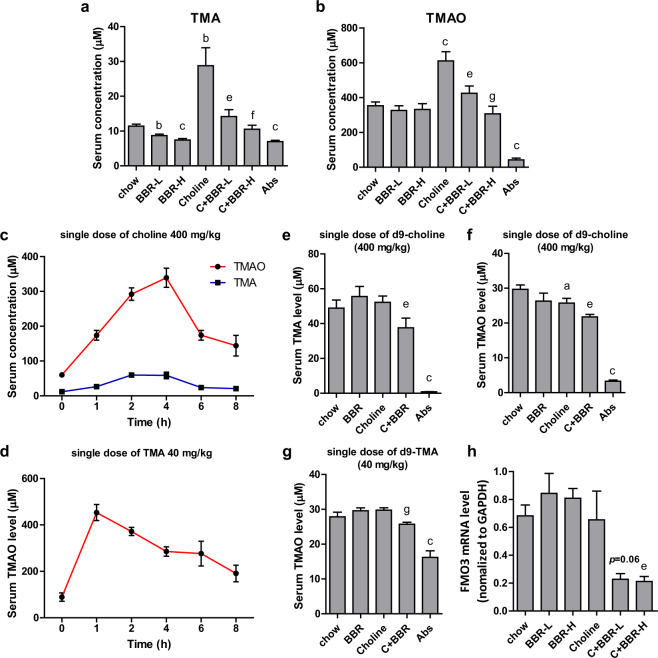
BBR decreased TMAO level in ApoE KO mice. **a**, **b** Eight-week-old female ApoE KO mice (*n* = 10 per group) were fed a chow or choline (1%) diet with or without BBR (BBR-L, 100 mg/kg; BBR-H, 200 mg/kg) and chow with Abs for 4 months. Serum TMA and TMAO levels were measured by HPLC/MS. **c**, **d** Eight-week-old female C57BL/6J mice were administered a single dose of choline (400 mg/kg, *n* = 10) or TMA (40 mg/kg, *n* = 10). Blood samples were collected at indicated times and serum TMA and TMAO levels were determined by HPLC/MS. **e**–**g** ApoE KO mice fed a chow diet or choline diet (1%) with or without BBR (100 mg/kg) for 4 months (*n* = 10 per group) were administered a single dose of d9-choline (400 mg/kg) or d9-TMA (40 mg/kg). At 4 h after choline was given, or 1 h after TMA was given, the mice were euthanized and blood was collected. Serum d9-TMA and d9-TMAO levels were determined by HPLC/MS. **h** Expression levels of FMO3 gene mRNAs were quantified using RT-qPCR assays. Values are presented as means ± SEM (*n* = 10). a *p* < 0.05; b *p* < 0.01; c *p* < 0.001 (versus chow-diet group); e *p* < 0.05; f *p* < 0.01; g *p* < 0.001 (versus choline-diet grou*p*).

To further investigate whether BBR-mediated TMAO reduction improved the pathologic changes of atherosclerosis under high choline condition, ApoE KO mice were given 1% choline in the chow diet for 4 months. After 4 months’ treatment of 100 or 200 mg/kg BBR (BBR-L or BBR-H), the body weight and food intake were similar between the groups. The serum TMA and TMAO levels under chow diet were 11.4 and 353.9 µM respectively, which were significantly increased by 2.5 and 1.7 folds with 1% choline addition. The choline diet-induced increments of TMA and TMAO levels were significantly decreased by BBR in a dose-dependent manner (Fig. 2a, b). Similarly, in chow diet-fed ApoE KO mice, BBR decreased the serum TMA level in a dose-dependent manner, while serum TMAO level appeared unaffected (Fig. 2a, b).

To further investigate the TMAO formation in mice that were treated with choline and BBR for 4 months, we detected the d9-TMAO contents in serum after administration of a single dose of d9-choline (400 mg/kg of body weight) or d9-TMA (40 mg/kg) by intragastric gavage. First, TMAO contents in serum were measured at 0, 1, 2, 4, 6, and 8 h after administration of a single dose of choline (400 mg/kg) or TMA (40 mg/kg) in the C57BL/6J mice. It was found that peak concentrations of TMAO with the treatment of choline or TMA occurred 4 or 1 h after respective administration (Fig. 2c, d). These times points were used for subsequent experiments. Following a single dose of d9-choline, both serum d9-TMA and d9-TMAO levels decreased significantly in BBR-treated mice in the choline diet-fed group, while barely any d9-TMA and d9-TMAO were produced in Abs treated mice (Fig. 2e, f). These results suggested that BBR treatment inhibited the metabolism of choline to TMA and thus TMAO through gut microbiota in choline diet-fed ApoE KO mice.

In addition, when BBR-treated mice were given d9-TMA, d9-TMAO levels were also lower than those in the control group under choline condition, but not altered under chow condition (Fig. 2g). This suggests that BBR may inhibit the transition of TMA to TMAO in the liver of ApoE KO mice fed choline. We then determined the level of FMO3 mRNA in the liver, and consistently, the result showed that BBR decreased FMO3 expression in choline-fed ApoE KO mice but not in the chow groups (Fig. 2h).

**Fig. 3:**
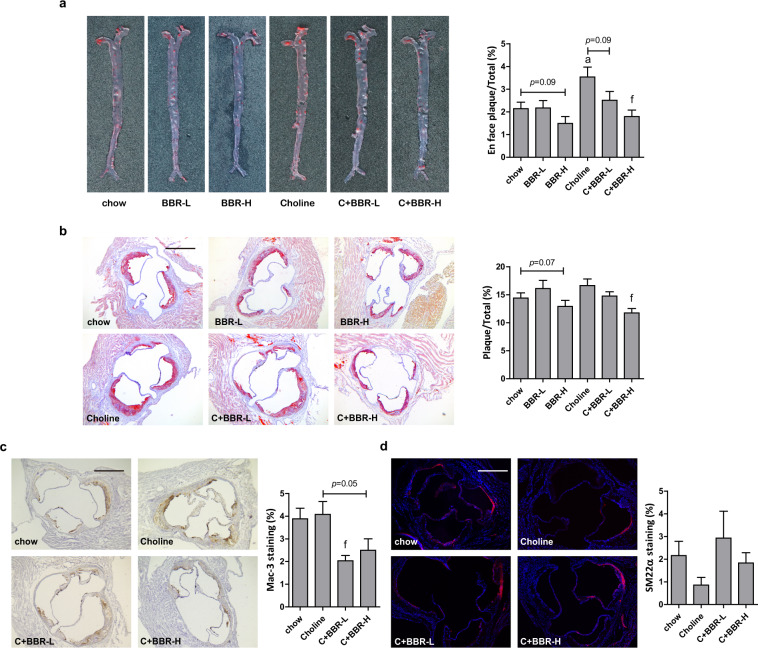
BBR protected ApoE KO mice from choline-induced atherosclerosis. Eight-week-old female ApoE KO mice (*n* = 10 per group) were fed with chow diet or choline diet in the absence or presence of 100 mg/kg BBR (BBR-L) or 200 mg/kg BBR (BBR-H) for 4 months. Atherosclerotic plaques in the whole aortas including the aortic arch, thoracic and abdominal regions (**a**), and aortic root (**b**) was assessed by Oil Red O staining, and the plaques area were quantified by ImageJ. **c**, **d** Immunostaining and fluorescence microscopy were applied to analyze the area of Mac-3-positive macrophages (**c**) and SM22α-positive smooth muscle cells (**d**) in the aortic root of ApoE KO mice. Scale bar = 500 μm. Representative images are shown. Values are presented as means ± SEM (*n* = 5–10). a *p* < 0.05 (versus chow-diet group); f *p* < 0.01 (versus choline-diet group).

Serum TMAO was reported to be tightly related to atherosclerosis^19^. Thus, we further investigated whether BBR-induced serum TMAO reduction was associated with the development of atherosclerosis in ApoE KO mice. Oil Red O (ORO) staining was used to visualize and assess atherosclerotic lesion development. As shown in Fig. 3a, b, choline markedly enhanced the atherosclerotic lesion areas in the whole aorta, and also increased lipid accumulation in the aortic root sections albeit not to a statistically significant level. All these effects were markedly reversed by BBR treatment in a dose-dependent manner (Fig. 3a, b). In chow diet-fed mice, 200 mg/kg BBR also slightly reduced the atherosclerotic plaques in both the whole aorta (*p* = 0.09) and aortic root sections (*p* = 0.07). Mac-3 was identified as a macrophage-specific biomarker of macrophages-derived foam cells. The results from an immunostaining assay showed that BBR treatment in choline diet markedly reduced mac-3 positive macrophages content in aortic roots (Fig. 3c), which was consistent with the effect on ORO-stained lesion size (Fig. 3b). In addition, under choline diet BBR treatment increased the level of SM22α-positive smooth muscle cells albeit just outside of statistical significance (Fig. 3d), which indicated plaque remodeling to a rather early and more stable stage, a hallmark of atherosclerosis retarding. Consistent with previous studies, the tendency of serum TMA and TMAO among different groups showed a significant positive correlation with that of plaque area by Linear Regression analysis (Supplementary Fig. 2). Taken together, with 1% choline feeding, BBR promoted the clearance of lipid deposits and overall decreased atherosclerotic progress along with decreasing serum TMAO level. However, in ApoE KO mice there was no change in serum total cholesterol (TC), LDL cholesterol (LDL-C), HDL cholesterol (HDL-C) or total bile acid (TBA) level in the BBR-treated group compared to the control group (Supplementary Fig. 3). These results suggested that decreasing TMAO originating from gut microbial metabolite TMA was the main cause of BBR’s atherosclerosis-protective role in this study. Also, BBR caused no damage to hepatic or renal function, as evidenced by no increase in serum levels of alanine transaminase (ALT), aspartate aminotransferase (AST), urea, creatinine (CRE) or total protein (TP). Even more, BBR markedly reversed choline diet-induced serum urea increment (Supplementary Fig. 3).

### BBR decreased TMA and TMAO levels and atherosclerosis via remodeling microbiota in ApoE KO mice

**Fig. 4:**
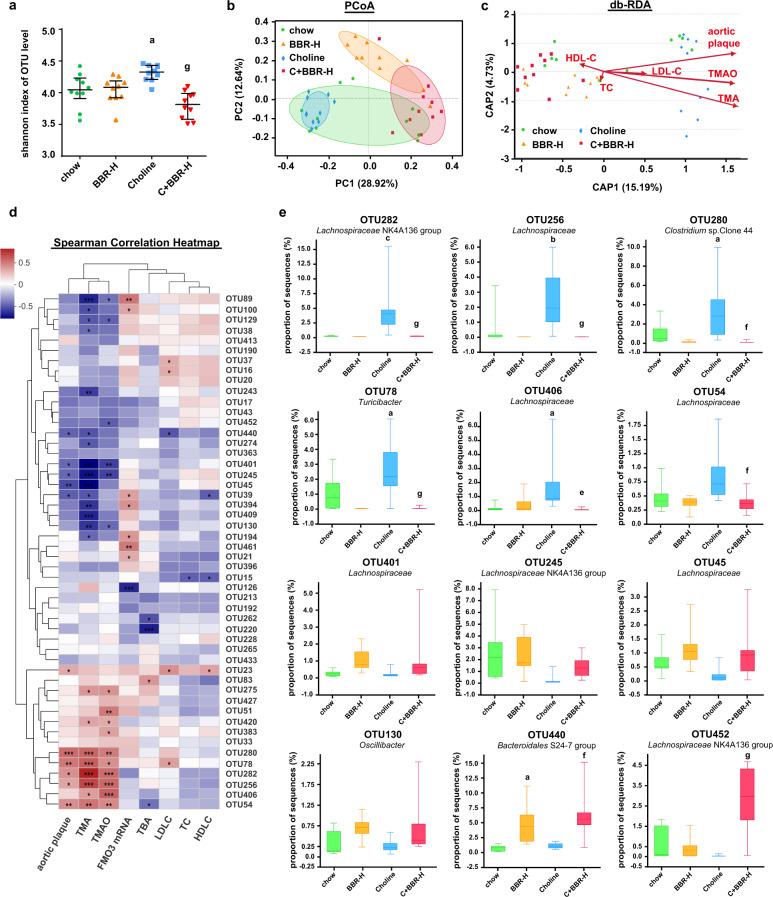
BBR inhibited TMA and TMAO production via remodeling gut microbiota in ApoE KO mice. Cecal content samples were collected from female ApoE KO mice which were fed a chow or choline diet for 4 months with or without BBR (*n* = 10 for each group), and 16S rRNA gene was amplified and sequenced. **a** Microbiota alpha diversity was measured using the Shannon index based on OTU level. Error bars were median with interquartile ranges, and *p* values were from Kruskal–Wallis *H* test. **b** PCoA analysis of bray curtis distance was analysis based on OTU level for microbiota beta diversity (ANOSIM *R* = 0.5659, *p* = 0.001). **c** Distance-based RDA showed the correlation between key microbiota phylotypes at OTU level and phenotypes (red arrows) including TMA (correlation coefficient *r*^2^ = 0.548, *p* = 0.001), TMAO (*r*^2^ = 0.3477, *p* = 0.001), aortic plaque area (*r*^2^ = 0.3477, *p* = 0.001), TC (*r*^2^ = 0.0132, *p* = 0.773), LDL-C (*r*^2^ = 0.0353, *p* = 0.502), and HDL-C (*r*^2^ = 0.0202, *p* = 0.705) within four groups based on the bray curtis algorithm. **d** Spearman correlation heat map demonstrated the correlation between key microbiota phylotypes and phenotypes (TMA, TMAO, aortic plaque area, TC, LDL-C, HDL-C, TBA and FMO3 mRNA) at the OTU level. The top 50 dominant OTU in all samples were selected to construct a heat map. Red denotes a positive association, blue a negative association, and white no association. *p < 0.05; **p < 0.01; ***p < 0.001. The annotations of the taxonomy of OTUs are in Supplementary Table 1. **e** The abundance of significant different OTUs designated in Fig. 4d among four groups. Boxes show the median with interquartile ranges (*n* = 10 for each group), whisker shows the minimum and maximum values. *p* values were from Kruskal–Wallis *H*. a *p* < 0.05; b *p* < 0.01; c *p* < 0.001 (versus chow-diet group); e *p* < 0.05; f *p* < 0.01; g *p* < 0.001 (versus choline-diet group).

To determine the role of the gut microbiota in the BBR-induced decrease of TMA and thus TMAO, we explored bacterial populations in the cecal contents of four groups ApoE KO mice (chow, BBR-H, Choline, C + BBR-H) by 16S rRNA gene sequence analysis (Supplementary Fig. 4a). An increased intestinal microbiota alpha diversity was observed in the choline group, which significantly decreased after 4 months of BBR treatment by using the Shannon index (Fig. 4a), the Sobs index, the Chao index or the Simpson index test (Supplementary Fig. 4b). The PCoA analysis showed that there were dramatic changes in microbiota composition among the four groups at OTU level (Fig. 4b). Through the LDA analysis, we found that at the genus level choline administration markedly enriched *Roseburia*, *Lachnospiracea*e (unclassified), *Alistipes*, *Turicibacter* etc. and BBR significantly increased the abundance of *Lachnospiraceae* NK4A136 group, *Bacteroidales* S24-7 group (unclassified), *Eubacterium* etc. (Supplementary Fig. 4c). Taken together, these results demonstrated that BBR altered the cecal microbial composition in ApoE KO mice.

In order to investigate the correlation between gut microbial composition remodeling and the BBR-induced phenotypic changes, we performed db-RDA analysis at OTU level. As shown in Fig. 4c, the cecal microbiota composition showed a notable correlation with both TMA and TMAO levels and with aortic plaque area. The Spearman’s correlation tests were applied at OTU level, and the results showed that more common OTUs were significantly correlated with plaque area and serum TMA/TMAO content, but not with TC, LDL-C, HDL-C, TBA or FMO3 mRNA levels (Fig. 4d). The abundance of OTU282, OTU256, OTU280, OTU78, OTU406, and OTU54 etc. were positively correlated with TMA and TMAO levels and with aortic plaque area, which was enriched in choline group and significantly decreased after BBR treatment (Fig. 4e). The abundance of OTU401, OTU245, OTU45, OTU130, OTU440, and OTU452 were negatively correlated with TMA and TMAO levels and with aortic plaque area, which was enriched after BBR treatment compared to the choline group (Fig. 4e). These results indicated that BBR played an important role in reducing serum TMA and TMAO levels and aortic plaque area by remodeling gut microbiota.

### BBR-modulated gut microbiome, especially *cutC* and *cntA* for TMA production

To further explore the mechanism of BBR to modulate gut microbiota, we performed metagenomic sequencing of cecal contents in 4 groups of C57BL/6J mice (chow, BBR-H, Choline, C + BBR-H, *n* = 21). An average of 49 million paired-end reads for each sample (ranging from 38 million to 60 million) was obtained, which led to a catalog of 3,118,451 nonredundant microbial genes. The C + BBR-H group (*n* = 5) had a notable reduction in gene counts (the number of genes identified per sample) compared to choline group (*n* = 6) (Supplementary Fig. 5a). This was consistent with the reduced community diversity of intestinal microbiota in 16S rRNA gene sequencing assays (Fig. 1c). On the basis of Bray-Curtis distances in PCoA assay, the overall structure of the gut microbiota from the four groups of mice showed significant alteration (Supplementary Fig. 5b). Sequences associated with four distinct domains were detected, and the majority microbial population were Bacteria (98.94–99.12%), with a low abundance of Eukaryota (0.31–0.48%), Archaea (0.06–0.08%) and Viruses (0.08–0.12%). Interestingly, the proportion of sequences assigned to Archaea and Viruses were significantly decreased in the C + BBR-H group compared to the choline group (Supplementary Fig. 5c–e). By LDA analysis at the genus level, the bacteria enriched in the choline group including *Clostridium*, *Eubacterium*, *Lachnoclostridium*, *Roseburia*, *Odoribacter* etc. and the C + BBR-H group significantly enriched *Bacteroides*, *Prevotella*, *Parabacteroides*, *Alloprevotella* etc. (Supplementary Fig. 5f), which is mostly consistent with the 16S rRNA gene sequencing results (Fig. 1e).

To explore the functional changes of the gut microbiota that might contribute to the improved phenotypic outcomes of BBR treatment, the non-redundant coding sequences were annotated based on the Kyoto Encyclopedia of Genes and Genomes (KEGG) database. The result revealed that differential enrichment of microbial metabolic pathways by BBR treatment was linked to carbohydrate, amino acid and energy metabolism (Supplementary Fig. 6a). The genes for carbohydrate utilization—carbohydrate-active enzyme (CAZy) showed similar PCoA patterns as that of the total genes (Supplementary Fig. 6b), suggesting that the carbohydrate utilization changes in the gut microbiota contributed to BBR treatment-induced protection from atherosclerosis. The bile salt hydrolase (BSH), a key enzyme involved in bile acid deconjugation^40^, decreased in the choline group and was restored by BBR treatment (Supplementary Fig. 6c). Accordingly, the ratio of T-β-MCA/β-MCA (conjugated/unconjugated β-muricholic acid) was significantly reduced in the BBR-treated mice compared to that in choline-fed mice, while the serum TBA level was not affected (Supplementary Fig. 6d).

**Fig. 5:**
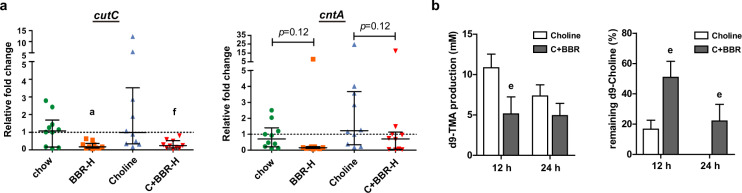
BBR regulated gut microbiota functional genes *cutC* and *cntA* for TMA production in ApoE KO mice. ApoE KO mice were fed choline diet for 4 months with or without BBR (*n* = 10 for each group). **a** The relative fold change of abundances of *cutC* and *cntA* in cecal content samples of ApoE KO mice were measured by qPCR, normalized to the 16S rRNA gene abundance from each sample. Error bars show the median with interquartile ranges using Kruskal–Wallis *H* test. **b** The intestinal microbiota of the cecal contents from choline-fed ApoE KO mice with or without BBR treatment (100 mg/kg) were used for an ex vivo assay. In the anaerobic fermentation, the transformation of d9-choline to d9-TMA was measured at 12 and 24 h, respectively. Values are presented as means ± SEM (*n* = 10). a *p* < 0.05 (versus chow-diet group); e *p* < 0.05; f *p* < 0.01 (versus choline-diet group).

In this study, TMA and TMAO levels decreased by BBR in choline-fed mice led us to focus on *cutC* and *cntA*, which are key genes responsible for TMA production^41^. However, according to the metagenomic sequencing data, there was a very low abundance of *cutC* gene (on average, 0.005%), and *cntA* gene was not even detected in any of the samples. This was consistent with the previous study^42^ and thus, quantitative PCR (qPCR) was applied to further measure the abundance of the two genes in cecal contents of ApoE KO mice using the degenerate primers designed by Rath et al.^42^. To verify the specificity and the quality of the qPCR results, amplified products were electrically separated on an agarose gel for visual inspections (Supplementary Fig. 7). Normalized to the 16S rRNA gene from each sample, the relative abundance of *cutC* and *cntA* genes was decreased after BBR treatment under both chow- and choline-diet conditions (Fig. 5a).

To further confirm the influence of BBR on the potential microbial communities responsible for TMA generation, cecal contents from choline-fed ApoE KO mice with or without BBR treatment were collected and cultured for an ex vivo choline-TMA transformation assay. The result showed that d9-TMA production was markedly reduced and the remaining d9-choline content was significantly increased in the BBR group compared to that in the choline group at both time points, suggesting that the transformation of d9-choline to d9-TMA in the anaerobic fermentation of the cecal contents was significantly decreased in BBR group (Fig. 5b). These results demonstrated that BBR could remodel the gut microbiome for inhibition of TMA production and ultimate TMAO formation in mice, especially by decreasing the levels of *cutC* and *cntA* genes.

### BBR inhibited TMA formation in TMA-producing strains

Considering the antibacterial activity of BBR, we explored whether the BBR-mediated reduction of TMA was due to its inhibitory effect on the growth of anaerobic strains. The minimum inhibitory concentration (MIC) of BBR against four reported TMA-producing human commensal strains (*Clostridium aparagiforme*, *Clostridium sporogenes*, *Anaerococcus hydrogenalis,* and *Escherichia fergusonii*) and four non-TMA-producing strains (*Clostridium perfringens*, *Escherichia coli*, *Bacteroides fragilis,* and *Bacteroides thetaiotaomicron*)^43^ was anaerobically tested using standard methods including the agar dilution method and the broth microdilution method^44^. From both methods, a similar result was found that BBR has different inhibitory effects on the growth of these strains (Table 1). Notably, BBR showed stronger suppression on TMA-producing bacteria *A. hydrogenalis* and *C. sporogenes* than other detected strains, with MIC of 64 and 128 µg/ml, respectively.

**Table 1:** Minimum inhibitory concentration (MIC) of BBR on human commensal bacteria under anaerobic condition.

	Strains	Agar dilution method		Broth microdilution method	
		MIC (µg/ml)^a^			
		MTR^b^	BBR	MTR^b^	BBR
TMA-producing strains (Community A)	*Clostridium asparagiforme* DSM 15981	0.5	512	>64	>512
	*Clostridium sporogenes* ATCC 19404	0.25	128	0.25–0.5	128
	*Anaerococcus hydrogenalis* DSM 7454	1	64	1–4	64
	*Escherichia fergusonii* ATCC 35469	>64	>512	>64	>512
non-TMA producing strains (Community B)	*Clostridium perfringens* CICC 22949	1–2	256	4	256
	*Escherichia coli* CGMCC 1.2385	>64	>512	>64	>512
	*Bacteroides fragilis* ATCC 25285	0.5	256	0.5–1	256–>512
	*Bacteroides thetaiotaomicron* ATCC 29741	2–4	256	1–2	256–512

^a^Three biological repeated experiments were carried out under different measurement methods.

^b^MTR, metronidazole, as a positive control drug.

**Fig. 6:**
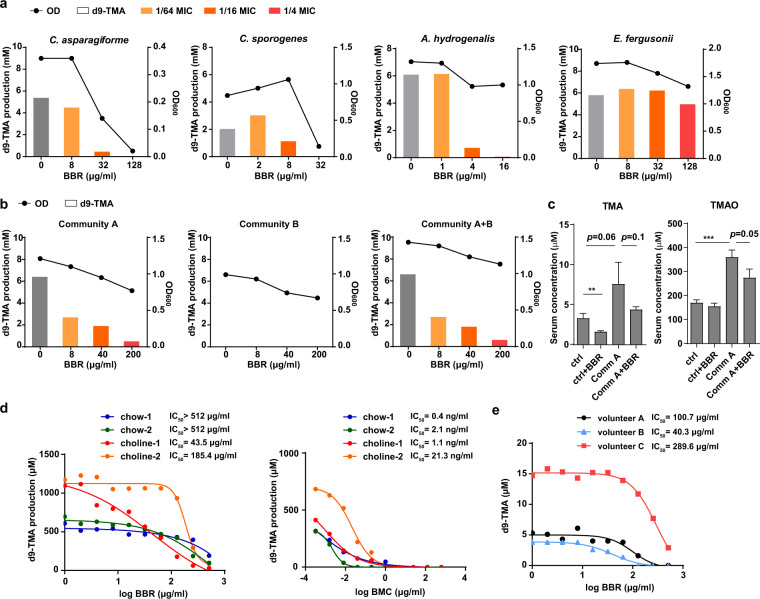
BBR inhibits anaerobic d9-TMA formation in vitro or ex vivo in a dose-dependent manner. **a** BBR inhibits d9-TMA formation from d9-choline in TMA-producing gut bacteria, including *C. aparagiforme*, *C. sporogenes*, *A. hydrogenalis,* and *E. fergusonii* under anaerobic condition. The production of d9-TMA was measured at 6 h by HPLC-MS/MS. The final concentrations of BBR were 1/64, 1/16, 1/4 of the MIC of each strain. **b** Effects of BBR on bacteria mixture under anaerobic condition. Four strains of TMA producing bacteria (Community A), four of non-TMA producing bacteria (Community B), and all eight (Community A + B) were cocultured with d9-choline for 3 h anaerobically. The d9-TMA productions were measured with HPLC-MS/MS. **c** BBR inhibits serum TMA and TMAO levels increased by colonization of TMA-producing bacteria. Choline supplemented chow-fed C57BL/6J mice were colonized with or without TMA-producing bacteria (Community A) and treated with or without BBR for 8 d. Values were presented as means ± SEM (**p < 0.01; ***p < 0.001; *n* = 5). **d** Effects of BBR on TMA-producing ability of mice gut microbiota under anaerobic condition ex vivo. The intestinal microbiota of the feces from chow or choline diet-fed C57BL/6J mice (*n* = 5 for each cage, the feces of the same cage were pooled) were used for the ex vivo assay. The production of d9-TMA was measured at 14 h. Dose-response curves were fit using nonlinear regression to determine IC_50_ values. BMC, bromomethylcholine. **e** BBR inhibits d9-TMA formation from d9-choline by human fecal suspension under anaerobic condition ex vivo. The fecal microbiota of three volunteer (A, B, C) were used for the ex vivo assay. The production of d9-TMA was measured at 6 h. Dose-response curves were fit using nonlinear regression to determine IC_50_ values.

Subsequently, the four TMA-producing strains were cultured anaerobically in vitro to assess the inhibitory effect of BBR on d9-TMA transformation from d9-choline. Considering its antibacterial activities, the working concentrations of BBR were set as 1/64, 1/16, 1/4 of the MIC of each strain. After 6 h of incubation, the TMA production was significantly inhibited by BBR at a concentration of 1/16 MIC and completely eliminated at 1/4 MIC, except for *E. fergusonii* (Fig. 6a). Especially at the concentration of 1/16 MIC, BBR markedly inhibited the choline-to-TMA transformation in the strains of *C. sporogenes* and *A. hydrogenalis* whereas their growth was hardly changed (Fig. 6a, Supplementary Fig. 8). To further discern whether BBR influenced TMA production independent of its antibacterial activity, we detected the effect of BBR on choline-to-TMA transformation in *C. sporogenes* cell lysate. The results showed that BBR treatment inhibited d9-TMA production at enzyme activity level in a dose-dependent manner (Supplementary Fig. 9), implying there are broader metabolic effects of BBR on gut microbiota other than its effect on the growth of gut microbes. Besides, we found BBR did not affect choline uptake in *C. sporogenes* at its non-lethal concentration (Supplementary Fig. 9).

Furthermore, to mimic the complex physiological environments of the microbial community in the intestinal tract, four TMA-producing strains (Community A, Table 1), four non-TMA-producing strains (Community B, Table 1), and the above eight strains (Community A + B, Table 1) were mixed. Cultures were incubated anaerobically with different concentrations (8, 40, and 200 µg/ml) of BBR and d9-choline for 3 h, then d9-TMA generation was detected. While Community B group could not produce TMA from choline as expected, in Community A and Community A + B groups BBR effectively inhibited TMA production in a dose-dependent manner without dramatically influencing the bacterial growth (Fig. 6b). These results showed that BBR could suppress TMA production from choline by some human intestinal bacteria at their sub-MIC anaerobically in vitro. To further demonstrate the role of TMA-producing microbes in BBR reduced TMAO content, TMA-producing strains from Community A were intragastrically administrated into C57BL/6J mice fed choline-supplemented chow diet with or without BBR treatment for 8 days. The results showed that the Community A transplant significantly increased serum TMAO level which was decreased by BBR (Fig. 6c), providing a direct evidence of the critical role of BBR in the inhibition of gut microbe-induced TMA and TMAO formation.

We next examined the effect of BBR on gut microbes recovered from feces of C57BL/6J mice fed chow or high-choline diet for 6 weeks in an ex vivo assay. As previously reported, the CutC/D specific inhibitor bromomethylcholine (BMC) displayed a potent inhibition in TMA production (IC_50_ = 160 nM (40 ng/ml) against human fecal cultures ex vivo)^26^ and was used as a positive control. In our assay, TMA production was inhibited by both BBR and BMC in a dose-dependent manner, while BMC exhibited a much stronger suppression of TMA generation in gut microbes of mice under both diets (IC_50_ = 0.4–21 ng/ml, Fig. 6d). However, BBR had a better TMA inhibitory effect in gut microbes from choline diet-fed mice (IC_50_ = 43.5 and 185.4 µg/ml) than that from chow diet-fed mice (IC_50_ > 512 µg/ml, Fig. 6d), implying that BBR may exert different inhibitory capacities on diverse intestinal microbiome under different conditions. Finally, to assess the inhibitory effect of BBR on choline-to-TMA transformation by human gut microbes, fresh stool samples of three volunteers were cultured with different concentrations of BBR for 6 h under anaerobic conditions. Although there are apparent differences in the conversion rate of choline to TMA by intestinal bacteria from different individuals, BBR efficiently inhibited the TMA production ex vivo at similar concentrations in choline diet-fed mice (Fig. 6e). Overall, the results from in vitro, ex vivo to in vivo studies demonstrated that BBR can suppress anaerobic production of TMA in both bacterial isolates and in the complex gut microbial community, which may be the main underlying mechanism of BBR in reducing serum TMA and TMAO levels.

## Discussion

**Fig. 7:**
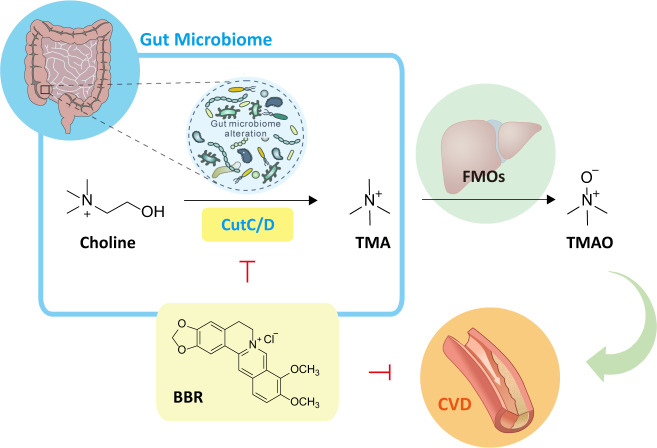
The role of BBR in the regulation of TMAO production and the treatment of CVD. BBR inhibits commensal microbial TMAO production via gut microbiota alteration. Black arrows indicate choline-TMA-TMAO transformation, green arrow represents promotion, and blocked line in red indicates inhibition.

BBR is abundantly found in the rhizomes of many Chinese traditional medicinal herbs, such as *Coptis chinensis* (Huang Lian in Chinese) which is widely used as an anti-diarrhea remedy in China for thousand years. The anti-diabetic activity of Huang Lian was documented for the first time in ~500 AD, and many contemporary pharmacological studies have demonstrated the therapeutic effects of BBR on cardiovascular diseases and metabolic disorders with good safety^28^. However, BBR exhibits a very low oral bioavailability which has been ascribed to the intestinal first-pass elimination^34^. During the last decade, accumulating evidences suggest a strong interaction between gut microbiota homeostasis and cardiovascular diseases or metabolic disorders, and some studies have been focused on the interaction of BBR with the gut microbiota, showing that BBR can alleviate obesity or atherosclerosis in high-fat diet-fed mice or rats via gut microbiota modulation^37,45,46^ and microbial metabolite production^37,38^. Recently, serum TMAO, a gut microbiota-derived metabolite mainly from animal protein-rich diets such as eggs, red meat, etc., was strongly associated with atherosclerosis in both human and animal trials^19,47,48^. There are some inconsistent reports^49^, but the discrepancy may be attributed to the different experimental setups including sex of the mice, diet background, and duration of choline feeding, etc^50^. A number of studies in mouse models suggest a causal relationship between plasma TMAO level and atherosclerosis^19,27,47,51^ and small molecule inhibitors of the major bacterial choline-TMA lyase have been reported to show efficacy to attenuate choline diet-enhanced atherosclerosis^25,26^. In this study, we disclosed the role and mechanism of the gut microbiota in BBR-induced protection against choline-induced atherosclerosis (Fig. 7).

It has been widely reported that BBR could reduce plasma cholesterol levels in ApoE or LDLR deficient mice fed a high-fat diet or Western diet^39,46,52–55^, which may influence cholesterol metabolism and bile acid metabolism significantly. Thus, to investigate the involvement of BBR in choline-TMA-TMAO pathway, choline-supplemented chow diet-fed mice were applied in this study to avoid the interference of cholesterol metabolism. In the atherosclerosis-prone ApoE KO mice, overdose of choline under chow diet took a longer period (16 weeks)^27,47^ to induce milder atherosclerosis than mice fed high cholesterol/fat diet (8–12 weeks). Oral administration of BBR effectively reduced the serum TMAO level in both choline-fed C57BL/6J and ApoE KO mice, and markedly limited progression of atherosclerotic plaque area in ApoE KO mice (Figs. 1–3). The mice had similar body weight and food intake, as well as serum TC, LDL-C, HDL-C or TBA levels and liver and renal function indexes (except that serum urea level was increased by choline supplementation and reversed by BBR treatment), in BBR-treated and untreated groups (Supplementary Fig. 3). The results suggest the serum TMAO level may be the major factor influencing the atherosclerosis severity in this study and BBR attenuates atherosclerosis by inhibition of TMA and TMAO production.

BBR treatment resulted in structural modulation of the gut microbiota in choline-supplemented chow diet-fed C57BL/6J and ApoE KO mice, with a significant reduction in the microbial diversity (Figs. 1, 4). This is consistent with some previous reports of BBR in high-fat diet-fed rats^36,45^. Although the overall gut microbiota richness was thought to be associated with healthy conditions in previous studies^56^, there are also some studies showed that this could not be always a reliable indicator of health in different situations^57^, especially at drug intervention. The decreased diversity of gut microbiota by BBR treatment may be ascribed to its known moderate to weak antibacterial activity against most tested bacteria^58^. We found BBR showed moderate growth inhibitory effects on TMA-producing bacteria under anaerobically incubation and may markedly inhibit the choline-to-TMA transformation in some strains even when their growth was hardly inhibited (Fig. 6a). More importantly, BBR may markedly decrease serum TMA and TMAO level increased by TMA-producing bacteria transplantation in choline-fed mice (Fig. 6c). But the mode of action of BBR on gut microbiota is quite different from Abs which exhibit potent anti-aerobe or anti-anaerobe activity. Indeed, the Abs would reduce the production of TMA more effectively (Figs. 1, 2) by inhibiting the growth of most intestinal bacteria (Supplementary Fig. 10), but may lead to severe gut microbiota dysbiosis and are not suitable for clinical long-term anti-atherosclerosis treatment. On the contrary, when repressing or killing harmful gut bacteria, BBR treatment enriched some beneficial gut bacteria significantly, which were negatively correlated with the TMA and TMAO levels as well as atherosclerotic plaque sizes (Fig. 4).

To gain an insight into the global gene diversity of gut microbiota influenced by choline and BBR, the analysis of the metagenomic data based on KEGG revealed differential enrichment of microbial metabolic pathways linked to carbohydrate utilization, however, the key genes involved in the SCFA production were not changed significantly in this study (Supplementary Fig. 6e) as in a previous study^37^, which may be attributed to the choline-supplemented chow diet used in this study instead of high-fat diet. Interestingly, it was observed that BBR restored the abundance of BSH gene as well as the ratio of conjugated/unconjugated β-muricholic acid which was changed in choline-fed mice, but the TBA level was not affected by choline addition or BBR treatment (Supplementary Fig. 6c and d). This was similarly found in the study of resveratrol which increased the BSH activity while inhibiting the production of TMAO and the progression of atherosclerosis^27^. Most importantly, the abundance of *cutC* and *cntA*, key genes responsible for two major pathways for TMA production, was decreased in BBR-treated mice (Fig. 5a). These results suggest that BBR may have various beneficial effects on gut microbiome functionality, especially the inhibition of TMA production in this study. If changes of gut microbiota functions other than choline-TMA-TMAO metabolism are involved in the role of BBR in high choline chow-fed mice remains to be investigated in future studies.

It is noteworthy that BBR showed a better TMA production inhibitory effect in gut microbes from choline-fed mice than that from chow diet-fed mice (Fig. 6d). Meanwhile, BMC, a microbial choline TMA lyase (CutC) specific inhibitor^26^, exhibited a much higher non-selective inhibitory effect on mice with both diets. The selective regulatory role of BBR in choline metabolism has also been shown in the in vivo animal experiments (Figs. 1, 2). The levels of TMA and TMAO only decreased significantly in choline-fed mice, but did not decrease or even slightly increased in chow-fed ApoE KO mice (Fig. 2) or C57BL/6J mice (Fig. 1), respectively. The result was also confirmed by an additional single-dose administration assay in C57BL/6J mice and this effect was abolished by Abs (Supplementary Fig. 11). This leads to an intriguing hypothesis that BBR may exert different inhibitory capacities on diverse intestinal microbiome, i.e., BBR may inhibit the formation of TMA by gut microbiota only when the choline has overdosed. If this is true, BBR will be a better modulator for TMA production by gut microbiota, as it will show different effects corresponding to different diets with different amounts of TMA-containing nutrient. The underlying mechanisms warrant further investigation.

Numerous clinical trials supported that BBR may inhibit the development of atherosclerotic plaque^59^. However, TMAO level was less investigated in BBR-treated patients. Notably, BBR showed a good inhibitory effect on TMA formation of gut microbiota from fecal samples of different volunteers (Fig. 6e). Given the different dietary habits, ages and health conditions, plasma TMA and TMAO levels showed great variation among human individuals as previously reported^60,61^. Although the plasma level of TMAO in mice was at least an order of magnitude higher than observed in humans^61^, the inhibitory effect of BBR on TMA formation of gut microbiota from human feces was comparable to that of choline-fed mice (Fig. 6d, e), at the concentration range of BBR (as high as 1–2 mg/g) detected in the gut content of different animals^62,63^. This result indicated that the effect and mechanism of BBR on TMA formation of gut microbiota observed here using choline-fed mice might be extrapolated to human. One clinical research in stable coronary artery disease patients found that the plasma TMAO level treated by BBR plus standard therapy (including aspirin and rosuvastatin) was 25 ± 16% lower after treatment, which was better than that of standard therapy alone (18 ± 16%), although did not reach statistical significance^64^. Further investigation will be needed to clarify the effect of BBR on TMAO level in humans, as both aspirin and statin were reported to reduce TMAO level in clinic studies^60,65^.

## Methods

### Animal and treatments

C57BL/6J mice (8 weeks, female) and ApoE KO mice with a C57BL/6 genetic background (8-weeks old, female) were purchased from Vital River Laboratory Animal Technology Co., Ltd (Beijing, China), and maintained at 22 ± 2 °C, 55 ± 5% relative humidity, with a 12-h light/dark period. In the experiments, mice were fed a standard chow diet (containing 0.1% choline) or choline diet (chow diet with 1% additional choline) with or without additional BBR (Deze biotechnology, Nanjing, China) and libitum access to water. A mixture of Abs (200 mg/kg vancomycin, 400 mg/kg neomycin sulfate, 400 mg/kg metronidazole, 400 mg/kg ampicillin; Sigma-Aldrich, St. Louis, MO, USA) were given by gavage. During the experiment, C57BL/6J mice and ApoE KO mice were fed for 6 weeks and 16 weeks before sacrificed respectively. Mice were weighed every week. At the end of the experiment, mice were anesthetized with pentobarbital sodium anesthesia (50 mg/kg of body weight) to surgical procedures to minimize suffering, then the heart was perfused with PBS. At euthanasia, tissues and cecal content were harvested and stored at −80 °C. All animal experiments were carried out in strict accordance with the recommendations in the Guide for the Care and Use of Laboratory Animals and were approved by the Institutional Authority for Laboratory Animal Care of Institute of Medicinal Biotechnology.

### HPLC-MS/MS detection of serum TMA, TMAO level

For quantitation of TMA, TMAO, d9-TMA and d9-TMAO level, serum protein was precipitated by adding 3-fold volumes of 80% acetonitrile at room temperature for 30 min. Samples were centrifuged (14,000 × *g*, 15 min, 4 °C) and the supernatants were filtrated and analyzed by HPLC-MS/MS. Briefly, HPLC-MS/MS analysis was carried out using an Agilent 1100 series HPLC coupled to Agilent 6410 Triple Quadrupole mass spectrometer (Agilent Technologies, Wilmington, DE, USA) equipped with an electrospray ionization source. Separation of TMA and TMAO was performed on an XBridge™ HILIC column (150 × 2.1 mm, the internal diameter of 3.5 μm; WATERS, Milford, MA, USA) with a flex capillary XBridge™ HILIC guard column (10 × 2.1 mm, the internal diameter of 3.5 μm; WATERS). The column was eluted isostatically at a flow rate of 0.25 ml/min with a mobile phase that mixed by an equal volume proportion of acetonitrile (phase A) and water with 10 mM ammonium formate (phase B). The capillary voltage was set at +4000 V and heated to 350 °C. Analytes were monitored in positive-ion mode with multiple reaction monitoring (MRM) of precursor and characteristic product-ion transitions of TMA at *m/z* 60 → 44, TMAO at m/z 76 → 58, d9-TMA at *m/z* 69 → 49 and d9-TMAO at *m/z* 85 → 66. Calibration curves were prepared by spiking various concentrations of the standard into control sample for quantification of serum analytes. The standards were purchased from Sigma-Aldrich (St. Louis, MO, USA).

### Evaluate the conversion rate of choline and TMA in vivo

To evaluate the peak TMA and TMAO concentrations in serum, the 8-week-old female C57BL/6J mice were made to fast overnight and given a dose of choline (400 mg/kg) or TMA (40 mg/kg) via gastric gavage. Blood samples were collected from the retro-orbital plexus at 0, 1, 2, 4, 6, and 8 h after gavage. Serum was collected and subjected to HPLC-MS/MS detection TMA and TMAO level as mentioned above.

ApoE KO mice fed a chow diet or 1% choline diet with or without BBR (100 mg/kg) for 4 months (*n* = 10 per group) were administered a single dose of d9-choline (400 mg/kg) or d9-TMA (40 mg/kg). At 4 h after choline was given, or 1 h after TMA was given, the mice were euthanized and blood was collected. Serum d9-TMA and d9-TMAO levels were determined by HPLC-MS/MS as mentioned above.

### Atherosclerotic plaque analysis

Atherosclerosis lesions were quantified in aortas by staining for lipid depositions with Oil Red O (ORO; Sigma-Aldrich) as described previously^66^. Briefly, for enface analysis of the aorta, aorta samples (from the proximal ascending aorta to bifurcation of the iliac artery) were dissected from mice and stored in 20% sucrose and cleaned from peri-adipose tissue after fixing in 4% paraformaldehyde for 48 h at 4 °C. Then the aortas were opened longitudinally and stained with ORO to determine the lesion area. For analyzing aortic root lipid deposition, the heart with ascending aorta was cut through below the aortic root, embedded in optimal cutting temperature (OCT) tissue freezing medium (Sakura Finetek, Torrance, CA, USA) following a snap frozen in liquid nitrogen and stored at −20 °C. The aortic sinus was sectioned by serial 7 µm slices (Leica CM1950; Leica Microsystems, Wetzlar, Germany), which were stained by ORO and pictured using Leica DM3000 Graphic Analysis System. All the pictures were analyzed and quantified using ImageJ software (U.S. National Institutes of Health, Bethesda, MD, USA). Atherosclerotic lesions were expressed as a percentage of total area, which was calculated by dividing the ORO-stained area over the total surface.

### Immunohistochemistry and immunofluorescence assay

Immunohistochemical staining was performed on the cryosections from the aortic root to characterize the presence of macrophages in the aortic sinus plaque with mouse macrophage-specific antibody (Mac-3) (BD Pharmingen, CA, USA) and pictured by Leica DM3000 Graphic Analysis System. Immunofluorescence staining was used to characterize the presence of smooth muscle cells (SMCs), determined with SM22α antibody (Abcam, Cambridge, UK) and Cy3-conjugated secondary antibodies (Abcam). Nuclei were labeled using DAPI and images were recorded using Olympus IX71 microscope (Olympus, Tokyo, Japan). All the primary antibodies and secondary antibodies were used in 1:100 dilution. All the images were processed and quantified with Leica Application Suite X (LAS X) microscope software (Leica Microsystems).

### Serum lipid analysis

Blood was collected by retro-orbital sampling. Serum TC, LDL-C, and HDL-C were enzymatically measured with the commercial cholesterol assay kit from Zhong Sheng Biotechnology (Beijing, China) according to the manufacturer’s instructions. Serum ALT and AST activity were also detected by kit from Zhong Sheng Bio-technology. All the detections were performed on Hitachi-7100 automatic analyser (Hitachi, Tokyo, Japan).

### Serum β-MCA and T-β-MCA level analysis

For β-muricholic acid (β-MCA) and tauro-β-muricholic acid (T-β-MCA) detection, 100 μl serum was added to 400 μl of methanol containing 0.1% formic acid. The sample was vortexed for 10 s and incubated at room temperature for 30 min. Then centrifuged at 14,000 × *g* for 10 min at 4 °C. The supernatant was transferred to a new tube and the organic phase was evaporated at 30 °C. The residue was reconstituted in 100 μl 50% methanol. Ten microliters of each reconstituted sample was injected for HPLC-MS/MS analysis. The chromatographic separation of bile acids was carried out on an Agilent 6410 Series Triple Quadrupole mass spectrometer. A Capcell Pak AQ C_18_ column (4.6 × 250 mm; internal diameter of 5.0 µm, Shiseido) was used to separate the bile acids. The HPLC conditions were: 60% B for 2 min, a gradient increasing to 100% B over 40 min, 100% B for 2 min, a gradient decreasing to 60% B over 10 min, and finally, 60% B for 10 min (solvent A = 2.6 mM ammonium acetate in water, pH 6.5; solvent B = methanol). The flow rate through the column was 0.8 ml/min. The detection and quantification of β-MCA and T-β-MCA were accomplished by MRM mode with the transitions of β-MCA at *m/z* 391.1 → 355.2 and T-β-MCA at *m/z* 516.3 → 462.4. Different concentrations of standards β-MCA (Sigma) and T-β-MCA (Sigma) were added to the control sample to generate calibration allowing to quantification.

### Quantification analysis of FMO3 by Quantitative real-time PCR

Total RNA was isolated from liver with the SV total RNA isolation system (Promega, Madison, WI, USA) and reverse transcription was preformed using the GoScript™ Reverse Transcription System (Promega) according to the manufacturer’s instructions. RNA and cDNA were quantified by Nanodrop spectrophotometry (Thermo Fisher Scientific, Waltham, MA, USA). Quantitative real-time PCR (qPCR) was performed in triplicate with the Bio-Rad CFX96 real-time system (Bio-Rad, Hercules, CA, USA) using the TaqMan^®^ Gene Expression Assays (Applied Biosystems, Foster City, CA, USA). The following primers and probes were used: flavin-containing monooxygenase 3 (FMO3; Fmo3; Mm01306345_m1), Glyceraldehyde-3-phosphate dehydrogenase (GAPDH; Gapdh; Mm99999915_g1) was used as the internal standard.

### Gut microbial metagenomic DNA extraction and microbial diversity analysis

Mice cecal content samples were collected and snap frozen at −80 °C. Total DNA was isolated and purified using the FastDNA® SPIN kit for Feces and the FastPrep® Instrument (MP Biomedicals, Santa Ana, CA, USA) according to the manufacturer’s instruction. The quality of DNA samples was assessed by gel electrophoresis and the concentration was measured by Nanodrop 8000 (Thermo Scientific, Wilmington, DE, USA). To investigate the microbiota community composition in the cecum, bacterial 16S ribosomal RNA (rRNA) gene sequencing assay was performed. Extracted DNA was used as a template to amplify the V4 region (for C57BL/6J mice) of the 16S rRNA gene with barcode-indexed primers 515F (5′-GTGYCAGCMGCCGCGGTAA-3′) and 806R1 (5′-GGACTACNVGGGTWTCTAAT-3′), and V3-V4 region (for ApoE KO mice) with barcode-indexed primers 338F (5′-ACTCCTACGGGAGGCAGCAG-3′) and 806R2 (5′-GGACTACHVGGGTWTCTAAT-3′). PCR was carried out with initial denaturation at 95 °C for 3 min and subsequently 27 cycles of denaturation at 95 °C for 30 s, annealing at 55 °C for 30 s, and extension at 72 °C for 45 s, and a final extension at 72 °C for 10 min. For each sample, three independent PCRs were performed to avoid bias. The PCR products for each sample were pooled and were extracted from a 2% agarose gel and further purified using the AxyPrep DNA Gel Extraction Kit (Axygen Biosciences, Union City, CA, USA). The amplicon libraries for high-throughput sequencing were subjected to the Illumina MiSeq platform according to standard protocols (C57BL/6J mice samples were performed by Promegene Technology Co., Ltd. (Shenzhen, China), and ApoE KO mice samples were performed by Majorbio Bio-Pharm Technology, Co., Ltd. (Shanghai, China). After removal of the barcodes and primers, the obtained sequences were clustered into the operational taxonomic units (OTUs) at a 97% similarity threshold and was aligned with SILVA128/16 S bacteria database for taxonomy information. Bioinformatics analysis of microbial diversity was analyzed on Majorbio I-Sanger Cloud Platform (www.i-sanger.com).

### Whole-metagenome shotgun sequencing and analysis

Metagenomic DNA was extracted from cecal content samples of C57BL/6J mice which were fed a chow or choline diet with or without BBR for 6 weeks by using the PowerSoil® DNA isolation kit (MoBio, Carlsbad, CA, USA) according to the manufacturer’s protocol. DNA was fragmented and sequenced in the Illumina HiSeq X Ten instrument at Promegene Technology Co., Ltd. Paired-end reads were decoded and trimmed. The remaining reads were filtered to eliminate host DNA based on the mice genome reference GRCm38.p5 and human genome reference GRCh38.p10. Clean reads were assembled into contigs using IDBA-UD software (http://i.cs.hku.hk/~alse/hkubrg/projects/idba_ud/) with default parameters. Genes were predicted by MetaGene (http://metagene.cb.k.u-tokyo.ac.jp). A non-redundant gene catalogue was constructed with CD-HIT (http://www.bioinformatics.org/cd-hit/), of which identity more than 95% with a minimum coverage over 90% were removed as redundancies. Gene taxonomy was annotated to NCBI NR databases by BLASTP search (version 2.2.28+, e-value cut-off 1e−5). Gene functional identification was annotated to KEGG (Kyoto Encyclopedia of Genes and Genomes, http://www.genome.jp/kegg/) by BLASTP search (version 2.2.28+, e-value cut-off 1e−5) and CAZy (Carbohydrate-active enzymes, http://www.cazy.org/) databases by hmmscan (e-value cut-off 1e−5), respectively. Principal coordinate analysis (PCoA), linear discriminant analysis (LDA) and relative abundance of domains/pathways/enzymes were analyzed on Majorbio I-Sanger Cloud Platform (www.i-sanger.com).

### Detection of *cutC*, *cntA* abundance via quantitative real-time PCR (qPCR)

The *cutC* and *cntA* gene abundances were measured by qPCR using degenerate primers (cutC_qF: 5′-TTYGCIGGITAYCARCCNTT-3′, cutC_qR: 5′-TGNGGYTCIACRCAICCCAT-3′; cntA_qF: 5′-TAYCAYGCITGGRCITTYAARCT-3′, cntA_qR: 5′-RCAGTGRTARCAYTCSAKRTAGTTRTCRAC-3′) designed by Rath et al.^42^. Amplification was performed with 100 ng of metagenomic DNA from cecal contents of ApoE KO mice using FS Universal SYBR Green Master (Rox, Roche, Germany) according to the manufacturer’s instructions. The final primer concentration was 400 nM. An initial 95 °C for 10 min was followed by 40 cycles of denaturation at 95 °C for 30 s; annealing at 60 or 53 °C for *cutC* or *cntA*, respectively, for 30 s; and an extension step at 72 °C for 20 s. Reactions were performed on the Bio-Rad CFX96 real-time system (Bio-Rad, CA, USA). The relative fold change of abundance levels was analyzed via the 2^−ΔΔCt^ method normalized to 16S rRNA gene levels from each sample using degenerate primers (16S_q515F: 5′-GTGCCAGCMGCCGCGG-3′, 16S_q806R, 5′-GGACTACHVGGGTWTCTAAT-3′) with an annealing temperature of 57 °C. qPCR amplified products were analyzed by agarose gel electrophoresis (5 μl each line).

### MIC test

Eight bacterial strains (see Table 1) were used in the susceptibility test for BBR. The MICs of BBR were determined through the agar dilution method and broth microdilution method according to the CLSI guidelines^44^. BBR was tested at concentrations of 1 to 512 µg/ml. Metronidazole was tested at concentrations of 0.125–64 µg/ml and used as positive control. *B. fragilis* and *B. thetaiotaomicron* were standard strains and served as quality controls. The MIC was defined as the lowest concentration of an agent that prevents turbidity after 48 h of anaerobic condition (10% CO_2_–10% H_2_–80 % N_2_) in AW400SG anaerobic workstation (Electrotek, West Yorkshire, UK). The experiment was repeated three times.

### Growth curve assay of TMA-producing bacteria with or without BBR treatment

TMA-producing bacteria, *Clostridium asparagiforme* DSM 15981, *Clostridium sporogenes* ATCC 19404, *Anaerococcus hydrogenalis* DSM 7454, and *Escherichia fergusonii* ATCC 35469 were inoculated in the 10 ml of Mega media^43^ with 15 mM d9-choline at an initial concentration of 1 × 10^6^ CFU/ml. The final concentrations of BBR were 1/64, 1/16, 1/4 of the MIC of each strain. Cultures were incubated for 0, 6, 9, 12, 24 h at 37 °C in anaerobic condition (10% CO_2_–10% H_2_–80% N_2_) using AW400SG anaerobic workstation (Electrotek). OD_600_ values were measured using Ultrospec 10 Cell Density Meter (Biochrom, Cambridge, UK).

### Quantitation of d9-TMA production from d9-choline in vitro

To determine the effects of BBR on TMA production in the above growth curves assay, cultures were harvested at 6 h (except for *C. asparagiforme*, which was harvested at 9 h).

To determine the effects of BBR on different bacteria mixture (see Table 1), four strains of TMA-producing bacteria (Community A), four of non-TMA-producing bacteria (Community B), and all eight (Community A + B) were mixed at 1 × 10^6^ CFU/ml of each strain and cocultured in 10 ml of Mega media with 15 mM d9-choline for 3 h anaerobically.

The production of d9-TMA in all culture medium samples was determined using HPLC-MS/MS according to the above-mentioned procedure.

### Colonization of C57BL/6J mice with TMA-producing bacteria

For in vivo study, four strains of TMA-producing bacteria (Community A) were anaerobically grown in Mega Medium for 24 h at 37 °C. Each strain was adjusted to 2–8 × 10^7^ CFU per mice. The supernatant was removed by centrifugation at 12,000 × *g* for 5 min and the strains were resuspended in saline and were combined, intragastrically administrated into C57BL/6J mice with or without BBR treatment for 8 days under choline diet condition. The mice were then sacrificed, and serum TMA and TMAO levels were determined using HPLC-MS/MS according to the above-mentioned procedure.

### Quantitation of d9-TMA production from d9-choline via ex vivo incubation

Fresh cecal content samples from choline-fed ApoE KO mice were diluted 1:20 (wt/vol) in sterile Mega medium. The dilution was vortexed for 1 min and centrifuged (100 × *g*, 30 s) at room temperature. A 50-μl supernatant was transferred to a capped 2-ml 96-deep-well plate containing 1 ml of Mega medium supplemented with 15 mM d9-choline. Samples were incubated in duplicate for 12 h, 24 h at 37 °C in anaerobic condition (10% CO_2_–10% H_2_–80% N_2_) using AW400SG anaerobic workstation (Electrotek). The concentration of d9-TMA in the culture medium was determined using HPLC-MS/MS according to the above-mentioned procedure. d9-choline was similarly quantified by HPLC-MS/MS using a Capcell-Pak C_18_ ADME column (250 × 4.6 mm, the internal diameter of 5.0 µm; Shiseido, Tokyo, Japan) at a flow rate of 1 ml/min with 98% (V/V) aqueous solution of 0.2% acetic acid (phase A) and 2% (V/V) methanol (phase B). *m/z* 113 → 69 for d9-choline.

To investigate the dose-response curves of BBR on TMA-producing ability of different diet-fed mice, the intestinal microbiota of the feces from chow or choline diet-fed C57BL/6J mice (*n* = 5 for each cage, the feces of the same cage were pooled) were cultured under anaerobic condition ex vivo. 50 mg of fresh sample was vortexed to suspended in 500 μL of sterile Brucella broth (BD Biosciences) and centrifuged (200 × *g*, 10 s, 4 °C). Then 100 μL of supernatant was inoculated into a capped 96-deep-well plate containing 1 ml of Brucella broth (containing 5% horse blood) supplemented with 1 mM d9-choline and BBR in doses ranging from 1 to 512 µg/ml. BMC was tested at doses ranging from 0.32 ng/ml to 625 µg/ml and used as the positive control. The plate was subsequently sealed with sterile foil and incubated anaerobically for 14 h at 37 °C. The concentration of d9-TMA in the culture medium was determined using HPLC-MS/MS according to the above-mentioned procedure. Dose-response curves were fit using nonlinear regression to determine IC_50_ values in Graphpad Prism software.

Similarly, the IC_50_ values of BBR on TMA-production were determined in human gut microbiota from three volunteers. Human fecal samples were collected from volunteers had not received Abs within 2 months of donation and provided written informed consent. 125 mg of fresh sample was vortexed to suspended in 1 mL of sterile Mega medium and centrifuged (200 × *g*, 10 s, 4 °C). Then 100 μL of supernatant was added into 1 ml of Mega medium supplemented with 1 mM d9-choline and BBR in doses ranging from 1 to 512 µg/ml. After anaerobic incubation at 37 °C for 6 h, the production of d9-TMA was measured by HPLC-MS/MS and IC_50_ values were calculated as described above-mentioned procedure.

### Determination of the effect of BBR on TMA production in *C. sporogenes* lysates

*C. sporogenes* ATCC 19404 was inoculated in 100 ml of sterile Mega media supplemented with 1 mM choline chloride (to induce expression of the *cut* genes) and grown anaerobically at 37 °C overnight. Cells were harvested by centrifugation and resuspended in 10 mL of PBS buffer supplemented with one Mini Protease Inhibitor Tablet (Pierce). Cells were placed in a Lysing Matrix B tube (MP Biomedicals) and then were disrupted using FastPrep® Instrument (MP Biomedicals) following parameters of speed 6.0 m/s for 40 s, two cycles. The soluble protein fraction of the cell lysate was collected by centrifugation at 12,000 × *g* for 20 min at 4 °C. The protein concentration of the clarified cell lysates was determined by a BCA protein assay kit (Thermo Fisher Scientific Co.). Lysate concentrations were diluted to 1.5 mg/mL with PBS buffer. *C. sporogenes* lysate were incubated with 20 mM of d9-choline in the presence or absence of different concentrations of BBR for 13 h at 37 °C in anaerobic condition (10% CO_2_–10% H_2_– 80% N_2_) in AW400SG anaerobic workstation (Electrotek). Sealed reaction mixtures were stopped by addition of three-fold volumes of 80% acetonitrile (containing 0.1% (v/v) formic acid). The production of d9-TMA in all reaction mixtures was determined using HPLC-MS/MS according to the above-mentioned procedure.

### Quantitation of choline uptake

To determine the effects of BBR on bacteria choline uptake, *C. sporogenes* ATCC 19404 was inoculated in sterile Mega media until an OD_600_ of around 0.5. Intact cells (1 ml) were incubated with different concentrations of d9-choline in the presence of different concentrations of BBR for 15 min at 37 °C in anaerobic condition (10% CO_2_–10% H_2_– 80% N_2_) in AW400SG anaerobic workstation (Electrotek). Then the supernatant was collected by centrifugation and the extracellular d9-choline was quantified by HPLC-MS/MS according to the above-mentioned procedure.

### Statistical analysis

Data from animal experiments were presented as means ± SEM. Statistical analysis among groups were tested by a two-tailed Student’s *t*-test or one-way analysis of variance, followed by Bonferroni’s correction as applicable (GraphPad Prism Software; Graph-Pad). *p* values are two-sided and < 0.05 denote statistical significance. Error bars denote SEM. *p* values for microbiota alpha diversity were conducted with the Kruskal–Wallis *H* test with false discovery rate (FDR) adjustment. *p* values for four groups’ comparation in the relative abundance of OTUs/domains/pathways/enzymes were conducted with Kruskal–Wallis *H* test with Tukey-kramer post hoc test.

### Reporting Summary

Further information on research design is available in the Nature Research Reporting Summary linked to this article.

## Supplementary information

Supplementary Information

Reporting Summary

## Data Availability

Raw sequence reads for all 16S rRNA gene amplicon sequencing datasets from both C57BL/6J mice and ApoE KO mice have been deposited to the NCBI Sequence Read Archive [http://www.ncbi.nlm.nih.gov/sra] and are accessible under BioProject No. PRJNA667196 and PRJNA667205, respectively. All other remaining relevant data are provided in the article, Supplementary information, or available from the corresponding author upon reasonable request.
