# Supplementary material for: Berberine attenuates choline-induced atherosclerosis by inhibiting trimethylamine and trimethylamine-N-oxide production via manipulating the gut microbiome
Source: NPJ Biofilms Microbiomes. 2021 Apr 16;7:36. doi: 10.1038/s41522-021-00205-8 (PMC8052457; doi:10.1038/s41522-021-00205-8)
Supplement: Supplementary file 1 — Supplementary Information [file 41522_2021_205_MOESM1_ESM.pdf]

Supplementary Materials for

Berberine Attenuates Choline-Induced Atherosclerosis by Inhibiting Trimethylamine and Trimethylamine-*N*-Oxide production via Manipulating the Gut Microbiome

Xingxing Li[1,a,b], Chunyan Su[1,a,b], Zhibo Jiang[a], Yuxin Yang[a], Yue Zhang[a], Mengxia Yang[a], Xiumin Zhang[a], Yu Du[a], Jin Zhang[a], Li Wang*[,a], Jiandong Jiang*[,a], Bin Hong*[,a,b]

[a]NHC Key Laboratory of Biotechnology of Antibiotics, [b]CAMS Key Laboratory of Synthetic Biology for Drug Innovation, Institute of Medicinal Biotechnology, Chinese Academy of Medical Sciences & Peking Union Medical College, No.1 Tiantan Xili, Beijing 100050, China

[1] These authors contributed equally to this work.

*To whom correspondence should be addressed.

Email: wangli_imb@163.com (Li Wang); jiang.jdong@163.com (Jiandong Jiang); binhong69@hotmail.com, hongbin@imb.pumc.edu.cn (Bin Hong).

Table of Contents

Supplemental Fig. 1 BBR treatment induce the gut microbiota composition changes in C57BL/6J mice. 3

Supplemental Fig. 2 Linear regression analysis of the correlation between serum TMA/TMAO level and plaque area in ApoE KO mice. 4

Supplemental Fig. 3 Serum physiological and biochemical indexes analysis of BBR treated ApoE KO mice. 5

Supplemental Fig. 4 BBR treatment induce the gut microbiota composition changes in ApoE KO mice. 6

Supplemental Fig. 5 BBR treatment altered the gut microbiota gene counts and composition in C57BL/6J mice via metagenomic sequencing. 7

Supplemental Fig. 6 BBR regulated gut microbiota functional genes in C57BL/6J mice. 8

Supplemental Fig. 7 BBR regulated gut microbiota functional genes *cutC* and *cntA* for TMA production in ApoE KO mice. 10

Supplemental Fig. 8 Growth curves of TMA producing bacteria with or without BBR treatment. 11

Supplemental Fig. 9 The effect of BBR on choline-TMA transformation in cell lysate of *C. sporogenes* and choline uptake in *C. sporogenes*. 12

Supplemental Fig. 10 Antibiotics induced severe gut microbiota dysbiosis in C57BL/6J mice. 13

Supplemental Fig. 11 BBR increased TMA and TMAO synthesis from choline in chow-diet fed C57BL/6J mice. 14

Supplemental Tab. 1 The annotations of the taxonomy of OTUs. 15

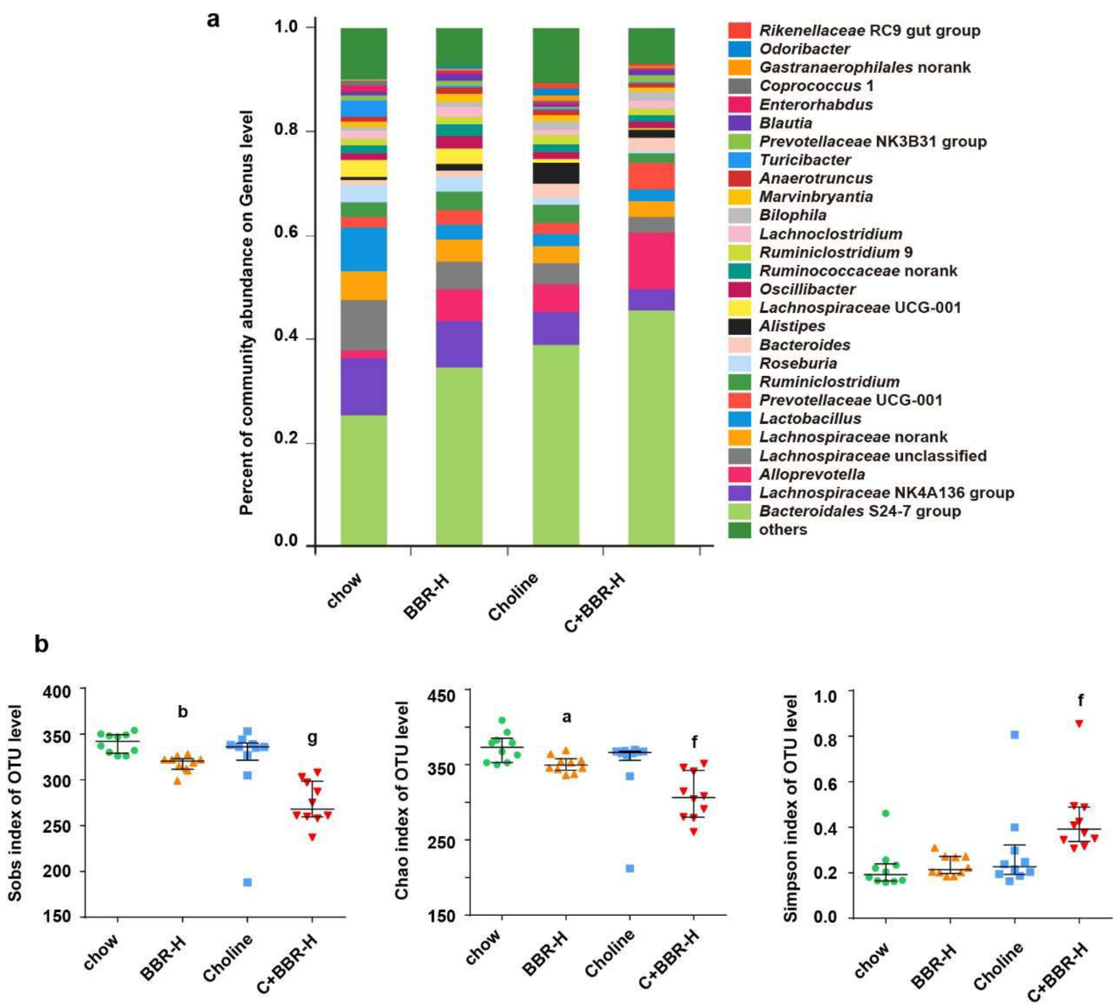

Supplemental Fig. 1 BBR treatment induce the gut microbiota composition changes in C57BL/6J mice.

Eight-week-old female C57BL/6J mice were fed chow, chow with 200 mg/kg BBR (BBR-H), chow with choline (1%), or chow with choline (1%) plus 200 mg/kg BBR (C+BBR-H) for 6 weeks. (**a**) Microbial composition in the cecal content samples from 4 groups of mice (chow, BBR-H, Choline, C+BBR-H) was measured by 16S ribosomal RNA (rRNA) gene sequence analysis. n=10 for each group. (**b**) Microbiota alpha diversity was measured by Sobs index, Chao index and Simpson index (based on OTU level). n=10 for each group. Error bars were median with interquartile ranges, and *p* values were from Kruskal-wallis *H* test. a, $p<0.05$; b, $p<0.01$ (versus chow-diet group); f, $p<0.01$; g, $p<0.001$ (versus choline-diet group).

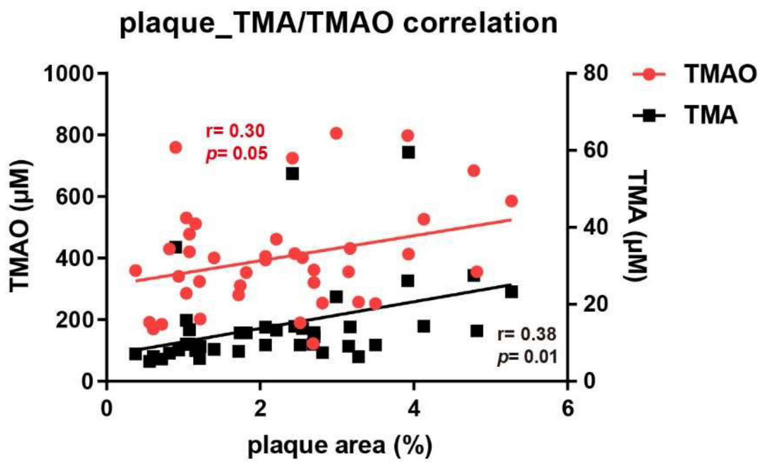

Supplemental Fig. 2 Linear regression analysis of the correlation between serum TMA/TMAO level and plaque area in ApoE KO mice.

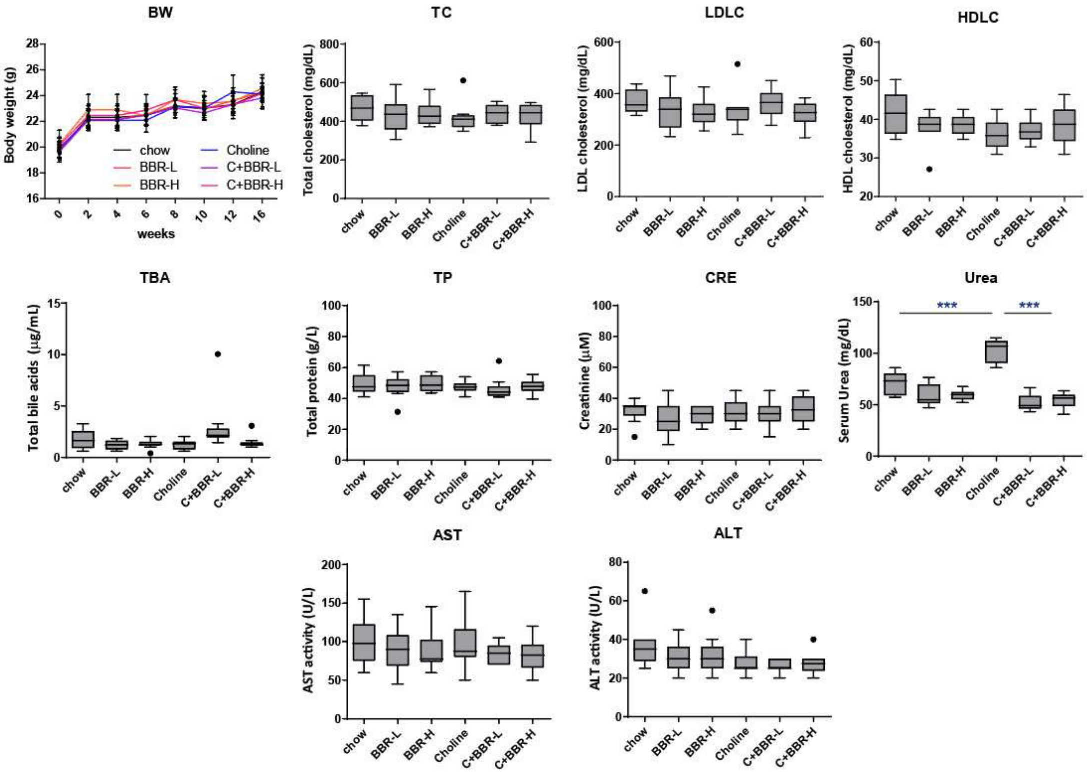

Supplemental Fig. 3 Serum physiological and biochemical indexes analysis of BBR treated ApoE KO mice.

Eight-week-old female ApoE KO mice (n=10 per group) were fed a chow or choline (1%) diet with or without BBR (BBR-L, 100 mg/kg; BBR-H, 200 mg/kg) for 4 months. Blood was collected and serum total cholesterol (TC), LDL cholesterol (LDLC), HDL cholesterol (HDLC), total bile acid (TBA), alanine transaminase (ALT), aspartate aminotransferase (AST), total protein (TP), creatinine (CRE) and Urea were analyzed using Hitachi-7100 automatic analyser. Data were shown as mean ± SEM. ***, p<0.001 (n=10).

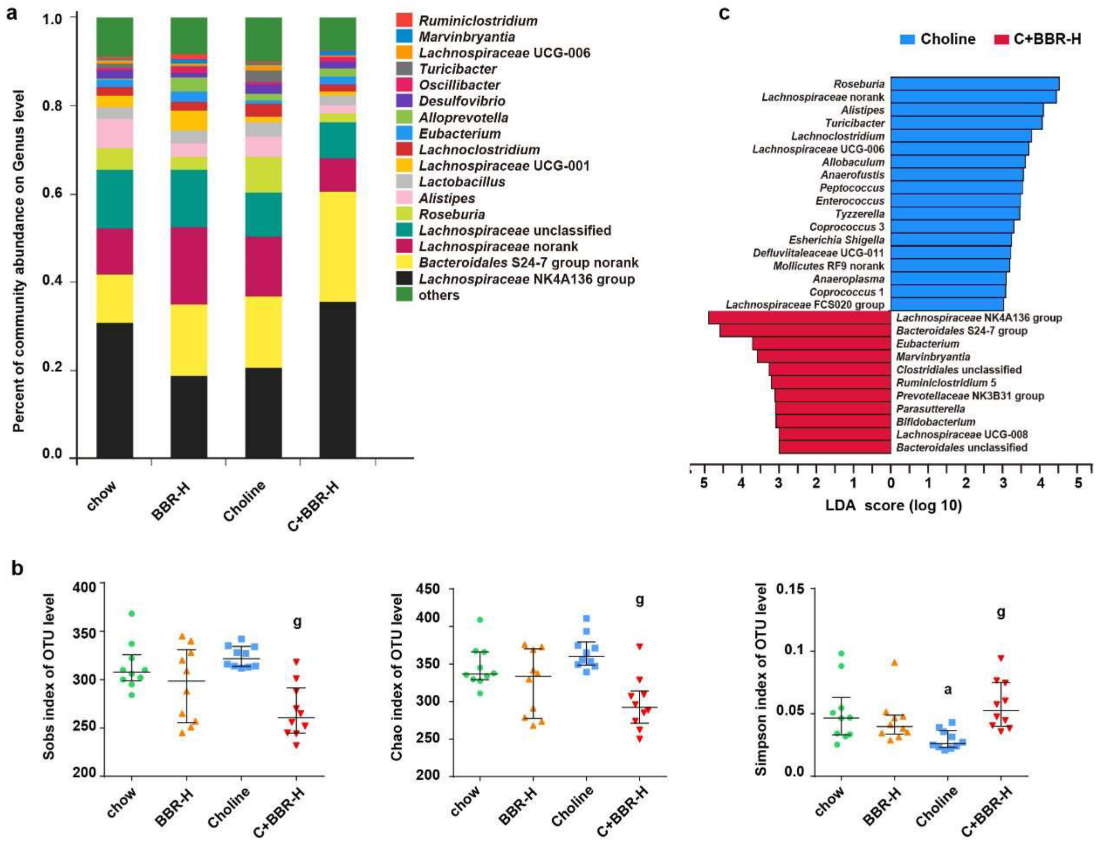

Supplemental Fig. 4 BBR treatment induce the gut microbiota composition changes in ApoE KO mice.

Eight-week-old female ApoE KO mice were fed chow, chow with 200 mg/kg BBR (BBR-H), chow with choline (1%), or chow with choline (1%) plus 200 mg/kg BBR (C+BBR-H) for 16 weeks. (**a**) microbial composition in the cecal content samples from 4 groups of mice (chow, BBR-H, Choline, C+BBR-H) was measured by 16S ribosomal RNA (rRNA) gene sequence analysis. n=10 for each group. (**b**) Microbiota alpha diversity was measured by Sobs index, Chao index and Simpson index (based on OTU level). n=10 for each group. Error bars were median with interquartile ranges, and p values were from Kruskal-wallis H test. a, $p<0.05$ (versus chow-diet group); g, $p<0.001$ (versus choline-diet group). (**c**) Linear discriminant analysis (LDA) identified the taxa most differentially abundant between the choline and C+BBR-H group at genus level. Only taxa meeting an LDA significant threshold value of ≥ 3.0 are shown.

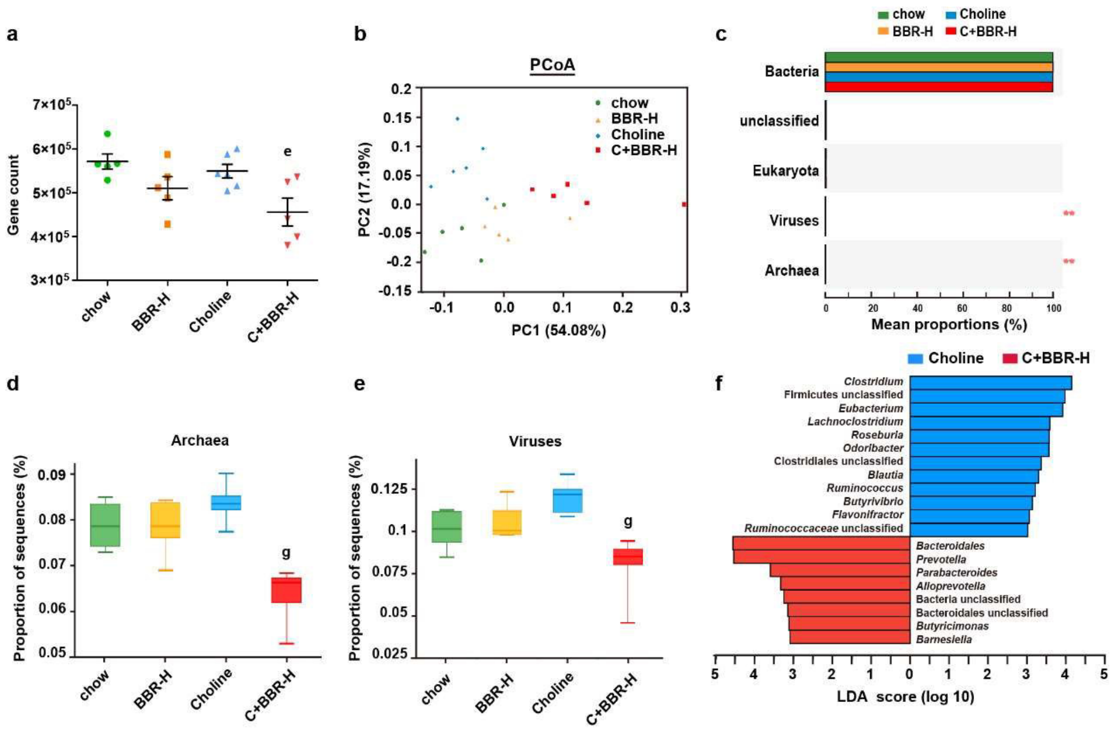

Supplemental Fig. 5 BBR treatment altered the gut microbiota gene counts and composition in C57BL/6J mice via metagenomic sequencing.

Cecal content samples from female C57BL/6J mice which were fed a chow or choline diet for 6 weeks with or without BBR were collected (n=5 for each group, except for Choline group n=6), and metagenomic DNA was extracted and sequenced. (**a**) The change in gene counts, which was adjusted to 38 million mapped microbial genes reads of per sample. Error bars were means ± SEM, and *p* values were from one-way ANOVA with the Tukey's multiple comparisons test. e, $p<0.05$ (versus choline-diet group). (**b**) PCoA analysis was performed on the basis of the bray curtis distance at species level for overall gut microbiota taxonomic compositions. (**c**) Relative abundance of genes involved in distinct domains. Boxes show the mean proportions. *p* values were from Kruskal-wallis *H* test with Tukey-kramer post hoc test. **, $p<0.01$. Changes in the relative abundance of Archaea (**d**) and Viruses (**e**) at domain level were analyzed. Boxes show the median with interquartile ranges, whiskers show the minimum and maximum values. *p* values were from Kruskal-wallis *H* test with Tukey-kramer post hoc test. g, $p<0.001$ (versus choline-diet group). (**f**) Linear discriminant analysis (LDA) identified the taxa most differentially abundant between the choline and C+BBR-H group at genus level. Only taxa meeting an LDA significant threshold value of ≥ 3.0 are shown.

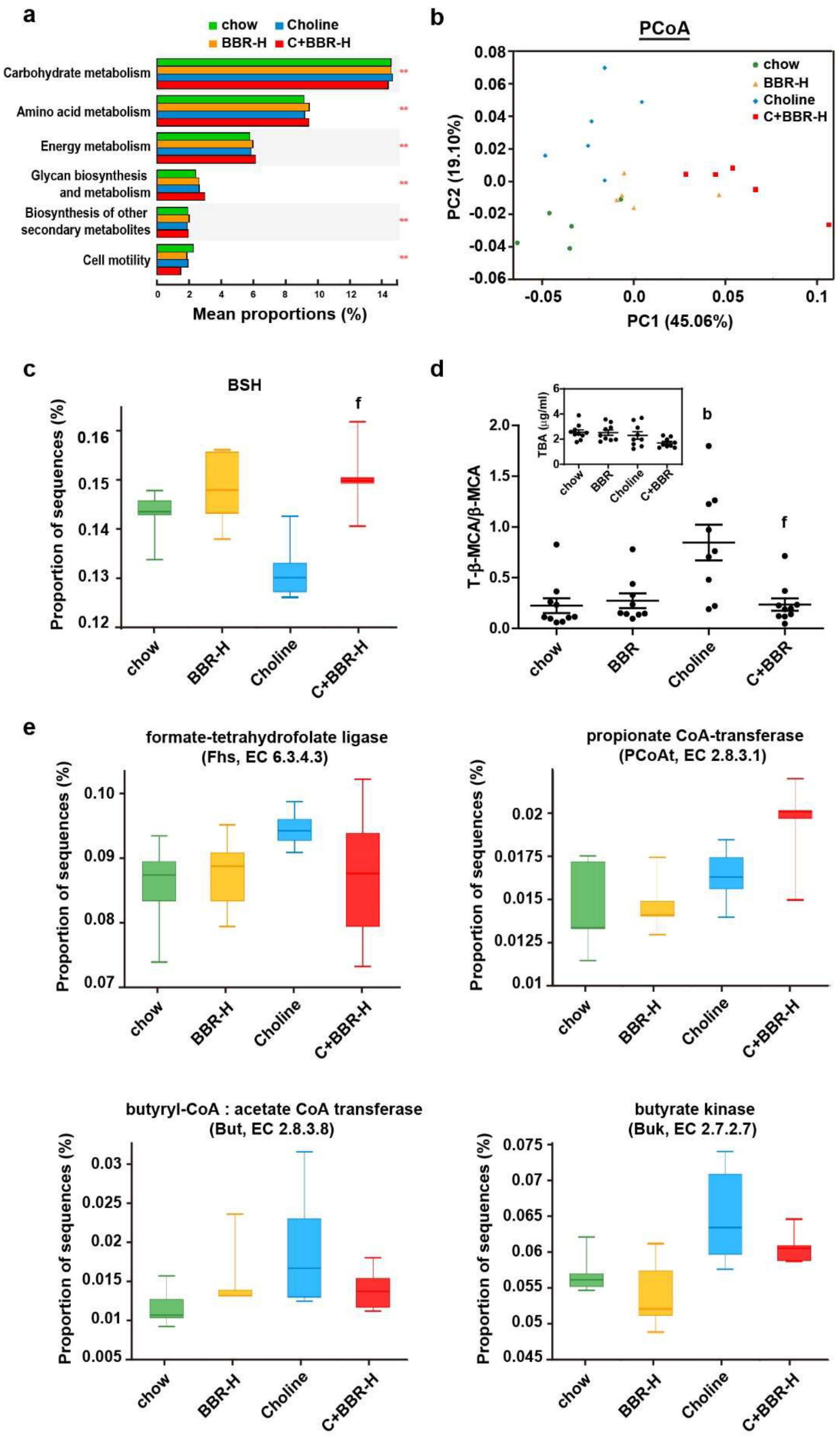

Supplemental Fig. 6 BBR regulated gut microbiota functional genes in C57BL/6J mice.

Cecal content samples from female C57BL/6J mice which were fed a chow or choline (1%) diet for 6 weeks with or without BBR were collected (n=5 for each group, except for Choline group

n=6), and metagenomic DNA was extracted and sequenced. (**a**) Relative abundance of genes involved in top six differential KEGG pathways. Boxes show the mean proportions. *p* values were from Kruskal-wallis H test with Tukey-kramer post hoc test. ** $p<0.01$. (**b**) PCoA was performed on the basis of the bray curtis distance for carbohydrate-active enzyme (CAZy) family-encoding genes. (**c**) Changes in the abundance of the bile salt hydrolase (BSH, EC 3.5.1.24) gene. Boxes show the median with interquartile ranges, whiskers show the minimum and maximum values. *p* values were from Kruskal-wallis *H* test with Tukey-kramer post hoc test. f, $p<0.01$ (versus choline-diet group). (**d**) TBA content and relative level of T-β-MCA/β-MCA in serum. C57BL/6J mice fed a chow diet or choline diet (1%) with or without BBR (100 mg/kg). Values are presented as means ± SEM (n=10). b, $p<0.01$ (versus chow-diet group); f, $p<0.01$ (versus choline-diet group). (**e**) Changes in the abundance of the key enzymes involved in SCFA production. formate-tetrahydrofolate ligase (Fhs, EC 6.3.4.3), propionate CoA-transferase (PcoAt, EC 2.8.3.1), butyryl-CoA:acetate CoA transferase (But, EC 2.8.3.8) and butyrate kinase (Buk, EC 2.7.2.7). Boxes show the median with interquartile ranges, whiskers show the minimum and maximum values. *p* values were from Kruskal-wallis *H* test with Tukey-kramer post hoc test. There were no significant difference between the groups with and without BBR treatment.

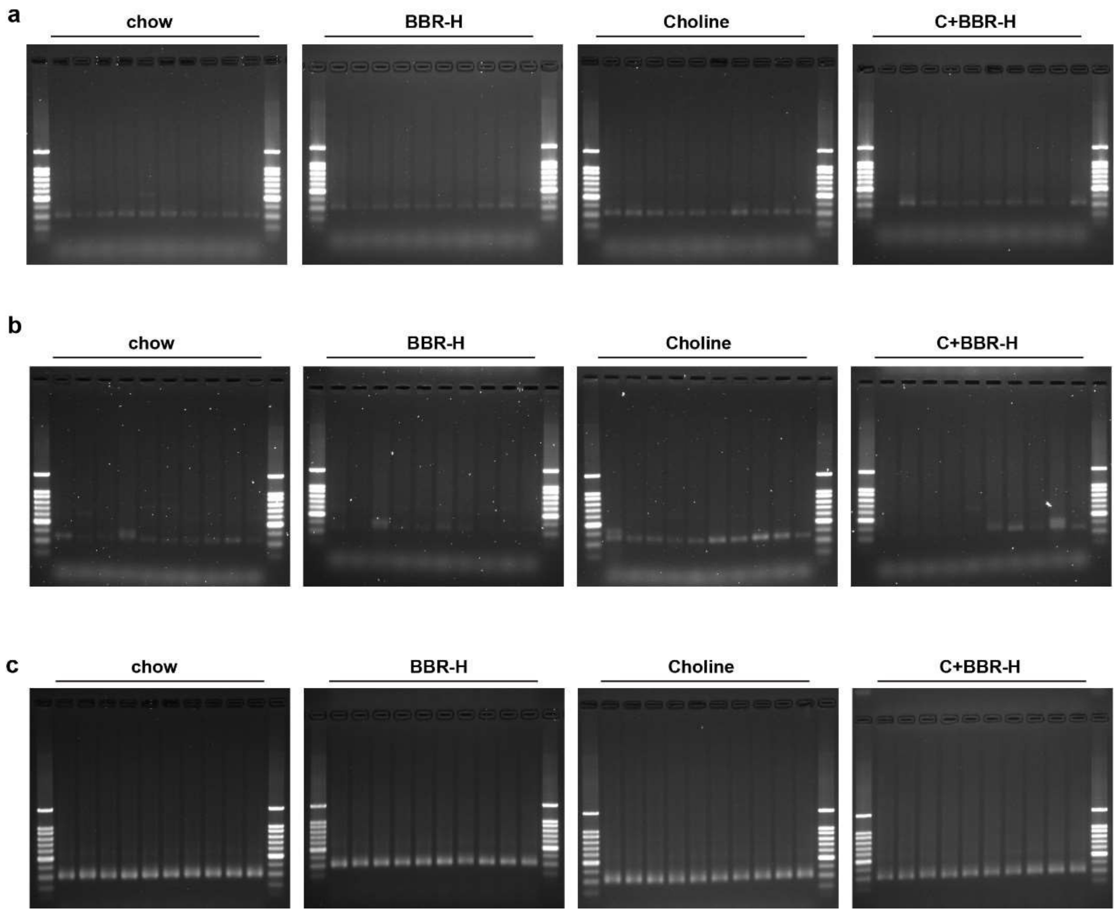

Supplemental Fig. 7 BBR regulated gut microbiota functional genes *cutC* and *cntA* for TMA production in ApoE KO mice.

mice were fed a chow or choline (1%) diet for 16 weeks with or without 200 mg/kg BBR (n=10 for each group). Metagenomic DNA was extracted and measured by qPCR. *cutC* (**a**), *cntA* (**b**) and 16S rRNA gene (**c**) amplified products were analyzed by agarose gel electrophoresis (5 µl each line). Expected sizes of amplified products are 315bp (*CutC*), 302bp (*cntA*) and 292bp (16S rRNA), respectively. DNA marker (from top): 1500 bp, 1000 bp, 900 bp, 800 bp, 700 bp, 600 bp, 500 bp, 400 bp, 300 bp, 200 bp, 100 bp. All gels derived from the same experiment and were processed in parallel.

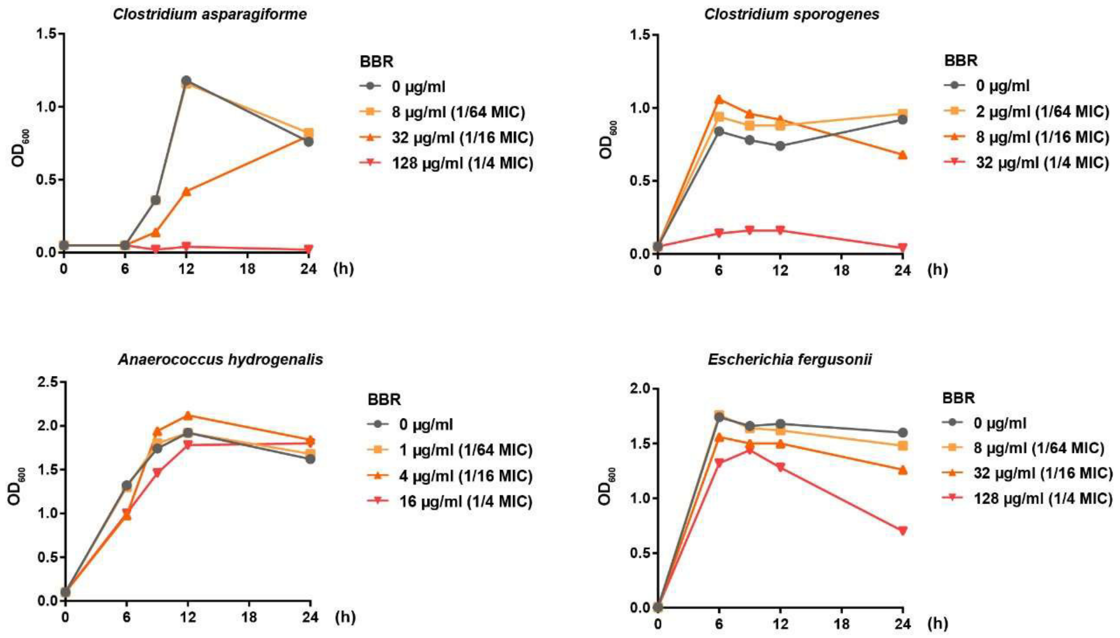

Supplemental Fig. 8 Growth curves of TMA producing bacteria with or without BBR treatment.

TMA producing bacteria cultures were incubated in Mega media containing 15 mM d9-choline with or without different concentration of BBR (1/64, 1/16, 1/4 of the MIC of each strain). The growth was detected at indicated time (0, 6, 9, 12 or 24 h) at 37 °C in anaerobic condition.

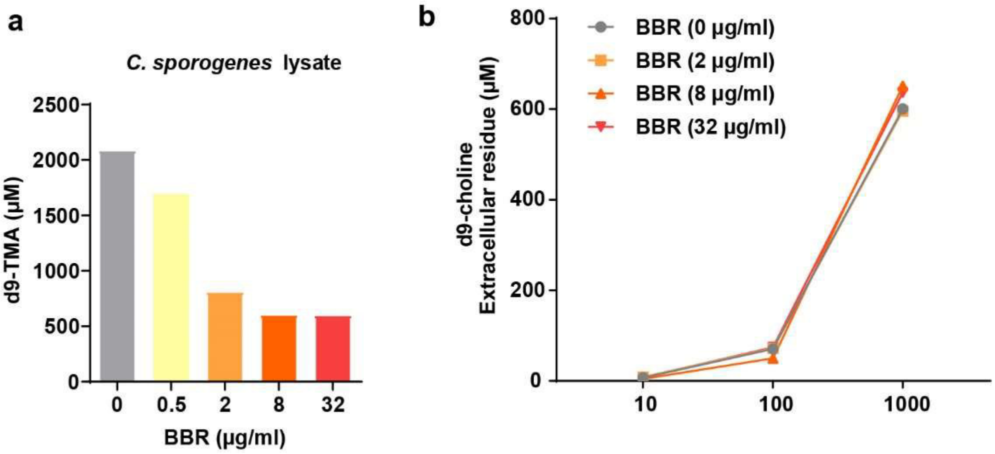

Supplemental Fig. 9 The effect of BBR on choline-TMA transformation in cell lysate of *C. sporogenes* and choline uptake in *C. sporogenes*.

(**a**) BBR inhibited d9-TMA production from d9-choline in *C. sporogenes* ATCC 19404 lysates. 1.5 mg/ml *C. sporogenes* lysates were incubated with 20 mM of d9-choline in the presence or absence of different concentrations of BBR for 13 hours at 37 °C in anaerobic condition. (**b**) BBR did not affect choline uptake in *C. sporogenes* ATCC 19404. Intact fresh strain (OD_{600} = 0.5) was incubated with indicated concentrations of d9-choline in the presence of different concentrations of BBR for 15 minutes at 37 °C in anaerobic condition. The cell exterior d9-choline was quantified.

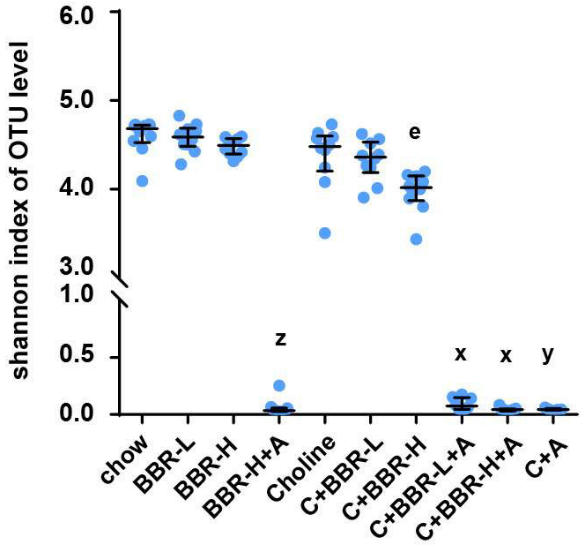

Supplemental Fig. 10 Antibiotics induced severe gut microbiota dysbiosis in C57BL/6J mice. Eight-week-old female C57BL/6J mice were fed chow, chow with 100 mg/kg BBR (BBR-L) or 200 mg/kg BBR (BBR-H), chow with choline (1%), or chow with choline (1%) plus 100 mg/kg BBR (C+BBR-L) or 200 mg/kg BBR (C+BBR-H) in the absence or presence of Abs (+A) for 6 weeks. Antibiotics mixture including 200 mg/kg vancomycin, 400 mg/kg neomycin sulfate, 400 mg/kg metronidazole and 400 mg/kg ampicillin. Microbiota alpha diversity was measured by 16S rRNA gene sequence analysis of the cecal content samples using Shannon index (based on OTU level). Error bars were median with interquartile ranges, and p values were from Kruskal-wallis H test (n=10). e, $p<0.05$ (versus choline-diet group); x, $p<0.05$; y, $p<0.01$; z, $p<0.001$ (versus relevant group without Abs).

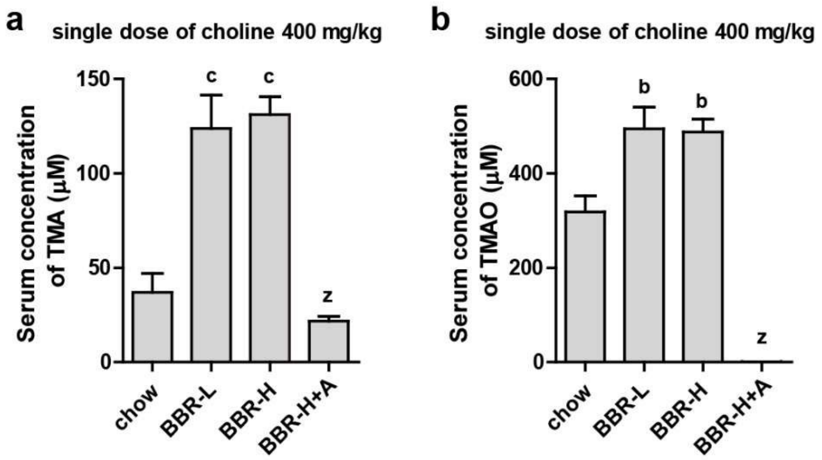

Supplemental Fig. 11 BBR increased TMA and TMAO synthesis from choline in chow-diet fed C57BL/6J mice.

Eight-week-old female C57BL/6J mice were fed a normal chow diet with or without BBR in the presence or absence of Abs for 6 weeks. Then the mice were administered choline (400 mg/kg, n=10). At 4 h after choline was given, blood samples were collected. Serum TMA (**a**) and TMAO (**b**) levels were determined by LC/MS. Values are presented as means ± SEM (n=10). b, p<0.01; c, p<0.001 (versus chow-diet group); z, p<0.001 (versus relevant group without Abs).

Supplemental Tab. 1 The annotations of the taxonomy of OTUs.

Domain	Kingdom	Phylum	Class	Order	Family	Genus	Species	OTU
d_Bacteria	k_norank	p_Firmicutes	c_Clostridia	o_Clostridiales	f_Lachnospiraceae	g_Lachnospiraceae_NK4A136_group	s_uncultured_bacterium_g_Lachnospiraceae_NK4A136_group	OTU89
d_Bacteria	k_norank	p_Firmicutes	c_Clostridia	o_Clostridiales	f_Lachnospiraceae	g_norank_f_Lachnospiraceae	s_unclassified_g_norank_f_Lachnospiraceae	OTU100
d_Bacteria	k_norank	p_Firmicutes	c_Clostridia	o_Clostridiales	f_Lachnospiraceae	g_unclassified_f_Lachnospiraceae	s_unclassified_f_Lachnospiraceae	OTU129
d_Bacteria	k_norank	p_Firmicutes	c_Clostridia	o_Clostridiales	f_Lachnospiraceae	g_Lachnospiraceae_UCG-001	s_unclassified_g_Lachnospiraceae_UCG-001	OTU38
d_Bacteria	k_norank	p_Firmicutes	c_Bacilli	o_Lactobacillales	f_Lactobacillaceae	g_Lactobacillus	s_Lactobacillus_johnsonii	OTU413
d_Bacteria	k_norank	p_Firmicutes	c_Clostridia	o_Clostridiales	f_Ruminococcaceae	g_Ruminiclostridium_9	s_unclassified_g_Ruminiclostridium_9	OTU190
d_Bacteria	k_norank	p_Firmicutes	c_Clostridia	o_Clostridiales	f_Lachnospiraceae	g_unclassified_f_Lachnospiraceae	s_unclassified_f_Lachnospiraceae	OTU37
d_Bacteria	k_norank	p_Bacteroidetes	c_Bacteroidia	o_Bacteroidales	f_Bacteroidales_S24-7_group	g_norank_f_Bacteroidales_S24-7_group	s_uncultured_Bacteroidales_bacterium_g_norank_f_Bacteroidales_S24-7_group	OTU16
d_Bacteria	k_norank	p_Bacteroidetes	c_Bacteroidia	o_Bacteroidales	f_Bacteroidales_S24-7_group	g_norank_f_Bacteroidales_S24-7_group	s_unclassified_g_norank_f_Bacteroidales_S24-7_group	OTU20
d_Bacteria	k_norank	p_Firmicutes	c_Clostridia	o_Clostridiales	f_Lachnospiraceae	g_norank_f_Lachnospiraceae	s_unclassified_g_norank_f_Lachnospiraceae	OTU243
d_Bacteria	k_norank	p_Firmicutes	c_Clostridia	o_Clostridiales	f_Lachnospiraceae	g_Lachnospiraceae_NK4A136_group	s_unclassified_g_Lachnospiraceae_NK4A136_group	OTU17
d_Bacteria	k_norank	p_Firmicutes	c_Clostridia	o_Clostridiales	f_Lachnospiraceae	g_Lachnospiraceae_NK4A136_group	s_uncultured_bacterium_g_Lachnospiraceae_NK4A136_group	OTU43
d_Bacteria	k_norank	p_Firmicutes	c_Clostridia	o_Clostridiales	f_Lachnospiraceae	g_Lachnospiraceae_NK4A136_group	s_uncultured_bacterium_g_Lachnospiraceae_NK4A136_group	OTU452
d_Bacteria	k_norank	p_Bacteroidetes	c_Bacteroidia	o_Bacteroidales	f_Bacteroidales_S24-7_group	g_norank_f_Bacteroidales_S24-7_group	s_uncultured_bacterium_g_norank_f_Bacteroidales_S24-7_group	OTU440
d_Bacteria	k_norank	p_Firmicutes	c_Clostridia	o_Clostridiales	f_Lachnospiraceae	g_[Eubacterium]_xylanophilum_group	s_uncultured_bacterium_g_[Eubacterium]_xylanophilum_group	OTU274
d_Bacteria	k_norank	p_Firmicutes	c_Clostridia	o_Clostridiales	f_Lachnospiraceae	g_Marvinbryantia	s_uncultured_bacterium_g_Marvinbryantia	OTU363
d_Bacteria	k_norank	p_Firmicutes	c_Clostridia	o_Clostridiales	f_Lachnospiraceae	g_unclassified_f_Lachnospiraceae	s_unclassified_f_Lachnospiraceae	OTU401
d_Bacteria	k_norank	p_Firmicutes	c_Clostridia	o_Clostridiales	f_Lachnospiraceae	g_Lachnospiraceae_NK4A136_group	s_uncultured_bacterium_g_Lachnospiraceae_NK4A136_group	OTU245
d_Bacteria	k_norank	p_Firmicutes	c_Clostridia	o_Clostridiales	f_Lachnospiraceae	g_unclassified_f_Lachnospiraceae	s_unclassified_f_Lachnospiraceae	OTU45

d_Bacteria	k_norank	p_Firmicutes	c_Clostridia	o_Clostridiales	f_Lachnospiraceae	g_Roseburia	s_uncultured_bacterium_g_Roseburia	OTU39
d_Bacteria	k_norank	p_Firmicutes	c_Clostridia	o_Clostridiales	f_Lachnospiraceae	g_norank_f_Lachnospiraceae	s_unclassified_g_norank_f_Lachnospiraceae	OTU394
d_Bacteria	k_norank	p_Bacteroidetes	c_Bacteroidia	o_Bacteroidales	f_Bacteroidales_S24-7_group	g_norank_f_Bacteroidales_S24-7_group	s_unclassified_g_norank_f_Bacteroidales_S24-7_group	OTU409
d_Bacteria	k_norank	p_Firmicutes	c_Clostridia	o_Clostridiales	f_Ruminococcaceae	g_Oscillibacter	s_[Clostridium]_leptum_g_Oscillibacter	OTU130
d_Bacteria	k_norank	p_Firmicutes	c_Bacilli	o_Lactobacillales	f_Lactobacillaceae	g_Lactobacillus	s_Lactobacillus_intestinalis	OTU194
d_Bacteria	k_norank	p_Firmicutes	c_Clostridia	o_Clostridiales	f_Lachnospiraceae	g_unclassified_f_Lachnospiraceae	s_unclassified_f_Lachnospiraceae	OTU461
d_Bacteria	k_norank	p_Bacteroidetes	c_Bacteroidia	o_Bacteroidales	f_Bacteroidales_S24-7_group	g_norank_f_Bacteroidales_S24-7_group	s_uncultured_Bacteroidales_bacterium_g_norank_f_Bacteroidales_S24-7_group	OTU21
d_Bacteria	k_norank	p_Bacteroidetes	c_Bacteroidia	o_Bacteroidales	f_Bacteroidales_S24-7_group	g_norank_f_Bacteroidales_S24-7_group	s_uncultured_bacterium_g_norank_f_Bacteroidales_S24-7_group	OTU396
d_Bacteria	k_norank	p_Bacteroidetes	c_Bacteroidia	o_Bacteroidales	f_Bacteroidales_S24-7_group	g_norank_f_Bacteroidales_S24-7_group	s_unclassified_g_norank_f_Bacteroidales_S24-7_group	OTU15
d_Bacteria	k_norank	p_Firmicutes	c_Clostridia	o_Clostridiales	f_Lachnospiraceae	g_Lachnospiraceae_NK4A136_group	s_unclassified_g_Lachnospiraceae_NK4A136_group	OTU126
d_Bacteria	k_norank	p_Bacteroidetes	c_Bacteroidia	o_Bacteroidales	f_Prevotellaceae	g_Alloprevotella	s_uncultured_Bacteroidales_bacterium_g_Alloprevotella	OTU213
d_Bacteria	k_norank	p_Bacteroidetes	c_Bacteroidia	o_Bacteroidales	f_Bacteroidales_S24-7_group	g_norank_f_Bacteroidales_S24-7_group	s_unclassified_g_norank_f_Bacteroidales_S24-7_group	OTU192
d_Bacteria	k_norank	p_Firmicutes	c_Clostridia	o_Clostridiales	f_Lachnospiraceae	g_Lachnospiraceae_NK4A136_group	s_uncultured_bacterium_g_Lachnospiraceae_NK4A136_group	OTU262
d_Bacteria	k_norank	p_Bacteroidetes	c_Bacteroidia	o_Bacteroidales	f_Bacteroidaceae	g_Bacteroides	s_Bacteroides_acidifaciens	OTU220
d_Bacteria	k_norank	p_Firmicutes	c_Clostridia	o_Clostridiales	f_Lachnospiraceae	g_norank_f_Lachnospiraceae	s_Lachnospiraceae_bacterium_A2	OTU228
d_Bacteria	k_norank	p_Firmicutes	c_Clostridia	o_Clostridiales	f_Lachnospiraceae	g_Lachnospiraceae_NK4A136_group	s_uncultured_bacterium_g_Lachnospiraceae_NK4A136_group	OTU265
d_Bacteria	k_norank	p_Firmicutes	c_Clostridia	o_Clostridiales	f_Lachnospiraceae	g_Lachnospiraceae_UCG-001	s_uncultured_bacterium_g_Lachnospiraceae_UCG-001	OTU433
d_Bacteria	k_norank	p_Bacteroidetes	c_Bacteroidia	o_Bacteroidales	f_Rikenellaceae	g_Alistipes	s_uncultured_bacterium_g_Alistipes	OTU23
d_Bacteria	k_norank	p_Proteobacteria	c_Deltaproteobacteria	o_Desulfovibrionales	f_Desulfovibrionaceae	g_Desulfovibrio	s_unclassified_g_Desulfovibrio	OTU83
d_Bacteria	k_norank	p_Firmicutes	c_Clostridia	o_Clostridiales	f_Lachnospiraceae	g_Lachnospiraceae_NK4A136_group	s_Lachnospiraceae_bacterium_A4	OTU275
d_Bacteria	k_norank	p_Firmicutes	c_Clostridia	o_Clostridiales	f_Lachnospiraceae	g_Lachnospiraceae_UCG-006	s_Clostridium_sp._ASF502	OTU427

d_Bacteria	k_norank	p_Firmicutes	c_Clostridia	o_Clostridiales	f_Lachnospiraceae	g_Lachnoclostridium	s_unclassified_g_Lachnoclostridium	OTU51
d_Bacteria	k_norank	p_Firmicutes	c_Clostridia	o_Clostridiales	f_Lachnospiraceae	g_Lachnospiraceae_NK4A136_group	s_uncultured_bacterium_g_Lachnospiraceae_NK4A136_group	OTU420
d_Bacteria	k_norank	p_Firmicutes	c_Clostridia	o_Clostridiales	f_Lachnospiraceae	g_Roseburia	s_unclassified_g_Roseburia	OTU383
d_Bacteria	k_norank	p_Firmicutes	c_Clostridia	o_Clostridiales	f_Lachnospiraceae	g_unclassified_f_Lachnospiraceae	s_unclassified_f_Lachnospiraceae	OTU33
d_Bacteria	k_norank	p_Firmicutes	c_Clostridia	o_Clostridiales	f_Lachnospiraceae	g_Roseburia	s_Clostridium_sp._Clone-44	OTU280
d_Bacteria	k_norank	p_Firmicutes	c_Erysipelotrichia	o_Erysipelotrichales	f_Erysipelotrichaceae	g_Turicibacter	s_uncultured_bacterium_g_Turicibacter	OTU78
d_Bacteria	k_norank	p_Firmicutes	c_Clostridia	o_Clostridiales	f_Lachnospiraceae	g_Lachnospiraceae_NK4A136_group	s_uncultured_bacterium_g_Lachnospiraceae_NK4A136_group	OTU282
d_Bacteria	k_norank	p_Firmicutes	c_Clostridia	o_Clostridiales	f_Lachnospiraceae	g_unclassified_f_Lachnospiraceae	s_unclassified_f_Lachnospiraceae	OTU256
d_Bacteria	k_norank	p_Firmicutes	c_Clostridia	o_Clostridiales	f_Lachnospiraceae	g_norank_f_Lachnospiraceae	s_uncultured_bacterium_g_norank_f_Lachnospiraceae	OTU406
d_Bacteria	k_norank	p_Firmicutes	c_Clostridia	o_Clostridiales	f_Lachnospiraceae	g_norank_f_Lachnospiraceae	s_unclassified_g_norank_f_Lachnospiraceae	OTU54
