# Supplementary material for: Berberine attenuates choline-induced atherosclerosis by inhibiting trimethylamine and trimethylamine-N-oxide production via manipulating the gut microbiome
Source: NPJ Biofilms Microbiomes. 2021 Apr 16;7:36. doi: 10.1038/s41522-021-00205-8 (PMC8052457; doi:10.1038/s41522-021-00205-8)
Supplement: Supplementary file 2 — Reporting Summary [file 41522_2021_205_MOESM2_ESM.pdf]

nature research

Corresponding author(s): Li Wang, Jiandong Jiang, Bin Hong

Last updated by author(s): Mar 15, 2021

Reporting Summary

Nature Research wishes to improve the reproducibility of the work that we publish. This form provides structure for consistency and transparency in reporting. For further information on Nature Research policies, see our Editorial Policies and the Editorial Policy Checklist.

Statistics

For all statistical analyses, confirm that the following items are present in the figure legend, table legend, main text, or Methods section.

n/a	Confirmed	
☐	☒	The exact sample size (*n*) for each experimental group/condition, given as a discrete number and unit of measurement
☐	☒	A statement on whether measurements were taken from distinct samples or whether the same sample was measured repeatedly
☐	☒	The statistical test(s) used AND whether they are one- or two-sided<br>*Only common tests should be described solely by name; describe more complex techniques in the Methods section.*
☒	☐	A description of all covariates tested
☐	☒	A description of any assumptions or corrections, such as tests of normality and adjustment for multiple comparisons
☐	☒	A full description of the statistical parameters including central tendency (e.g. means) or other basic estimates (e.g. regression coefficient) AND variation (e.g. standard deviation) or associated estimates of uncertainty (e.g. confidence intervals)
☒	☐	For null hypothesis testing, the test statistic (e.g. *F*, *t*, *r*) with confidence intervals, effect sizes, degrees of freedom and *P* value noted<br>*Give P values as exact values whenever suitable.*
☒	☐	For Bayesian analysis, information on the choice of priors and Markov chain Monte Carlo settings
☐	☒	For hierarchical and complex designs, identification of the appropriate level for tests and full reporting of outcomes
☒	☐	Estimates of effect sizes (e.g. Cohen's *d*, Pearson's *r*), indicating how they were calculated

Our web collection on statistics for biologists contains articles on many of the points above.

Software and code

Policy information about availability of computer code

Data collection	Collection of mass spectrometry data was performed using Mass Hunter Version B.08.00.
Data analysis	Most data analysis was performed using Graphpad Prism 7.<br>16S rRNA gene amplicon sequencing datasets and whole-metagenome shotgun sequencing datasets were analyzed on Majorbio I-Sanger Cloud Platform (www.i-sanger.com), as detailed in the methods section.

For manuscripts utilizing custom algorithms or software that are central to the research but not yet described in published literature, software must be made available to editors and reviewers. We strongly encourage code deposition in a community repository (e.g. GitHub). See the Nature Research guidelines for submitting code & software for further information.

Data

Policy information about availability of data

All manuscripts must include a data availability statement. This statement should provide the following information, where applicable:

- Accession codes, unique identifiers, or web links for publicly available datasets
- A list of figures that have associated raw data
- A description of any restrictions on data availability

Raw sequence reads for all 16S rRNA gene amplicon sequencing datasets from both C57BL/6J mice and ApoE KO mice have been deposited to the NCBI Sequence Read Archive [http://www.ncbi.nlm.nih.gov/sra] and are accessible under BioProject No. PRJNA667196 and PRJNA667205, respectively.
All materials are available from the indicated commercial sources or upon request to the corresponding author.

Field-specific reporting

Please select the one below that is the best fit for your research. If you are not sure, read the appropriate sections before making your selection.

☒ Life sciences ☐ Behavioural & social sciences ☐ Ecological, evolutionary & environmental sciences

For a reference copy of the document with all sections, see nature.com/documents/nr-reporting-summary-flat.pdf

Life sciences study design

All studies must disclose on these points even when the disclosure is negative.

Sample size	No statistical methods were used to predetermine sample size. In vivo mice studies suggested a group size of at least 5.
Data exclusions	No data were excluded from the analysis.
Replication	Replicate experiments were performed for all studies as noted in figure legends, methods, and Source Data. Experimental findings were reliably reproduced.
Randomization	Mice in all studies were randomized to their particular groups at time of allocation to experimental groups.
Blinding	Investigators performing quantitative analyses of mice serum assays were blinded to group allocation with samples labeled by code only. Investigators were not blinded to mouse group allocation during the performance of animal husbandry requirements for experiments.

Reporting for specific materials, systems and methods

We require information from authors about some types of materials, experimental systems and methods used in many studies. Here, indicate whether each material, system or method listed is relevant to your study. If you are not sure if a list item applies to your research, read the appropriate section before selecting a response.

Materials & experimental systems

n/a	Involved in the study	
☐	☒	Antibodies
☒	☐	Eukaryotic cell lines
☒	☐	Palaeontology and archaeology
☐	☒	Animals and other organisms
☒	☐	Human research participants
☒	☐	Clinical data
☒	☐	Dual use research of concern

Methods

n/a	Involved in the study	
☒	☐	ChIP-seq
☒	☐	Flow cytometry
☒	☐	MRI-based neuroimaging

Antibodies

Antibodies used	Anti-Mac-3 (CD107b) antibody: BD Pharmingen™, Catalog No. 550292, Clone M3/84, Lot 3179590.<br>Anti-SM22 alpha antibody: abcam, Catalog No. ab14106, Polyclonal, Lot GR157642-1
Validation	Anti-Mac-3 (CD107b) antibody<br>Species: Rat<br>Application: Flow cytometry (Routinely Tested), Immunohistochemistry-paraffin, Immunohistochemistry-zinc-fixed (Tested During Development), Immunohistochemistry-frozen, Immunoprecipitation (Reported)<br>Detailed in https://www.bdbiosciences.com/us/reagents/research/antibodies-buffers/immunology-reagents/anti-mouse-antibodies/cell-surface-antigens/purified-rat-anti-mouse-cd107b-m384/p/550292<br><br>Anti-SM22 alpha antibody<br>Species: Rabbit<br>Application: ICC/IF, WB<br>Detailed in https://www.abcam.com/taglntransgelin-antibody-ab14106.html

Animals and other organisms

Policy information about studies involving animals; ARRIVE guidelines recommended for reporting animal research

Laboratory animals	Eight-week-old female C57BL/6J mice & Eight-week-old female ApoE KO mice
Wild animals	the study did not involve wild animals.

Field-collected samples	the study did not involve samples collected from the field.
Ethics oversight	All animal experiments were carried out in strict accordance with the recommendations in the Guide for the Care and Use of Laboratory Animals and were approved by the Institutional Authority for Laboratory Animal Care of Institute of Medicinal Biotechnology.

Note that full information on the approval of the study protocol must also be provided in the manuscript.
